# Non-canonical signaling pathway of SNAI2 induces EMT in ovarian cancer cells by suppressing miR-222-3p transcription and upregulating PDCD10

**DOI:** 10.7150/thno.43198

**Published:** 2020-04-27

**Authors:** Lili Fan, Han Lei, Sai Zhang, Yulong Peng, Chunyan Fu, Guang Shu, Gang Yin

**Affiliations:** 1 Department of Pathology, Xiangya Hospital, School of Basic Medical Sciences, Central South University, Changsha, Hunan Province, China; 2 School of Basic Medical Sciences, Central South University, Changsha, Hunan Province; 3 China-Africa Research Center of Infectious Diseases, School of Basic Medical Sciences, Central South University, Changsha, Hunan Province, China

**Keywords:** Ovarian cancer, PDCD10, miR-222-3p, SNAI2, Migration

## Abstract

**Background**: Epithelial ovarian cancer (EOC) is one of the most lethal malignancies in women worldwide. Many studies showed the transcription factor SNAI2-induced Epithelial-Mesenchymal Transition (EMT) through inhibiting E-cadherin (E-cad) expression. Our previous study reported that miR-222-3p was an important tumor-suppressive miRNA for EOC development and dissemination. The present study aimed to acquire a deeper mechanistic understanding of the role of miR-222-3p regulation that might contribute to improving current anti-metastasis strategies in EOC.

**Methods**: A variety of techniques were used to measure mRNA and protein expression levels, including quantitative real-time polymerase chain reaction (qRT-PCR), Western blot, immunohistochemical (IHC) staining, and immunofluorescence (IF). Four different microRNA (miRNA) target prediction databases were used to predict the target genes of miR-222. Luciferase assay was performed to determine the direct binding of miR-222-3p to the untranslated region (3'-UTR) of PDCD10. The biological effects of PDCD10 and miR-222-3p were also investigated* in vitro* by Transwell and wound healing assays, as well as* in vivo* by a xenograft mice model. Combining UCSC and JASPAR, as well as ENCODE public databases, we predicted that the transcription factor SNAI2 could affect miR-222-3p expression. Luciferase assay was utilized to examine the validity of putative SNAI2 binding sites for miR-222-3p regulation. Chromatin immunoprecipitation (ChIP) was used to explore the SNAI2's occupancy on the miR-222-3p promoter.

**Results**: We observed the inhibitory effect of SNAI2 on miR-222-3p transcription and confirmed the tumor-suppressive function of miR-222-3p both in EOC cells and tissues. PDCD10 was upregulated and inversely correlated with miR-222-3p, both *in vitro* and* in vivo*, which was consistent with the information in bioinformatics databases. Furthermore, We observed direct binding of miR-222-3p to the 3'-UTR of PDCD10 and inhibition of PDCD10 translation, which, in turn, inhibited EOC cell migration* in vitro* and repressed EOC xenografted tumor metastasis* in vivo*. We found that genetic overexpression of PDCD10 (OE-PDCD10) increased cancer metastasis by down-regulating E-cad and enhancing Vimentin (VIM) thereby inducing EMT and promoting *β*-catenin/Wnt-mediated cell migration.

## Introduction

Epithelial ovarian cancer (EOC), which generally presents at an advanced stage in the most lethal malignancies in women, is the most common cause of gynecological cancer death 1. The major reason for the high mortality rate is because of the invasion of cancer cells into adjacent organs or metastasis to the peritoneal cavity 2. Nevertheless, the underlying molecular mechanisms that lead to EOC cell metastasis are not clear. Clarifying the mechanism of the invasion or metastasis in OC cells may contribute to the development of effective metastasis-targeted therapies and improve the overall survival (OS) of OC patients 3.

Accumulating evidence suggested that the metastatic ability in EOC cells was accompanied by the loss of cell-cell adhesion and gain of migratory and invasive traits in a process known as Epithelial to Mesenchymal Transition (EMT) 4. Several processes, such as tissue regeneration, embryonic development, and organ fibrosis, as well as tumor cell metastasis are governed by EMT 5. An important hallmark of EMT is decreased expression of E-cadherin (E-cad) 6. Besides E-cad, activation of the transcription factor SNAI2 and increased expression of vimentin (VIM) have also been found to act as critical factors in implicating EMT during tumor progression 7. Moreover, the Wnt/*β-*catenin pathway is regarded as the EMT switch of endothelial cells 8 and its activation is critical for tumor development 9. Endothelial signaling by *β-*catenin is critical during tumor progression 10 and high levels of *β-*catenin signaling may cause tumor cell migration and tumor metastasis 11. Nevertheless, our understanding of the molecular events that drive EMT signaling during tumor progression is incomplete. A better understanding of molecular mechanisms driving tumor metastasis would be helpful in exploiting new candidate genes for tumor therapy 7. It is well known that SNAI2 can inhibit E-cad expression and thus induce EMT 12, 13. However, it is not clear if SNAI2 could induce EMT by other signaling pathways.

It is well established that miRNAs are involved in post-transcriptional mechanisms for gene regulation in various physiological and pathological conditions 14. We previously reported that miR-222-3p could inhibit tumor cell proliferation by targeting GNAI2 in epithelial ovarian cancer 15. It has also been described that miR-222-3p could promote cell proliferation and tumor metastasis by targeting ERα in endometrial carcinoma 16. Although miR-222 has been shown to play a vital role in cancer metastasis 17, it has been suggested that miRNAs had specific activities during tumorigenesis of various cancers and/or in cancer stages that could even be antagonistic 18. However, the exact mechanism of miR-222-3p in suppressing cell migration in EOC is not clear yet.

Programmed cell death 10 (PDCD10), also known as krev-interaction trapped 5 (KRIT5) and cerebral cavernous malformations 3 (CCM3) 19, is one of the CCM family of proteins 20. It has been reported that the CCM family proteins KRIT1, CCM2, and PDCD10 are critical factors of endothelial cell-cell association and vascular equilibrium 21. A previous study revealed that PDCD10-MST4 interactions stabilize PDCD10 and MTS4 proteins and that PDCD10 promotes cell proliferation and migration that are dependent on MST4 kinase activity 22. Further research indicated that PDCD10 connects with the germinal center kinase III (GCKIII) kinases (involved in STK24 and STK25) 23. PDCD10 has been shown to be upregulated in pancreatic adenocarcinomas 24 and involved in resistance to cisplatin-induced apoptosis in metastatic colon cancer cells 25. However, the underlying mechanisms of action that led to these phenotypic features have not been fully elucidated.

In this study, we identified PDCD10 as an essential factor in EMT and Wnt/*β-*catenin signaling. Our data revealed that miR-222-3p could target PDCD10 and inhibit its translation thereby impeding EOC cell migration *in vitro* and repressing EOC xenografted tumor metastasis *in vivo*. Furthermore, we provided evidence that SNAI2 could inhibit the transcription of miR-222-3p and examined the role of the SNAI2/miR-222-3p/PDCD10 axis in EOC cells. Our data uncovered a new regulatory pathway involving SNAI2 to induce EMT and increase EOC metastasis.

## Materials and Methods

### Tissue specimens

Thirty-eight tumor tissue specimens along with 16 adjacent healthy tissues were obtained from EOC patients from the department of gynecological oncology, Hunan Cancer Hospital, between April 2013 and January 2018. All patients or patients' families signed the written informed consent, and the procedure of tissue collection was approved by the Ethics Review Committee of Xiangya Hospital, Central South University. After resection of the tumor and adjacent tissues, the specimens were immediately frozen in liquid nitrogen and stored at -80 ℃. All experiments strictly adhered to the code of ethics of the World Medical Association and were conducted following the guidelines of Central South University.

### Cell culture and transfection

The human fimbrial epithelial cell line FE25, the epithelial ovarian cancer cell lines A2780 (serous cystadenocarcinoma), HO 8910 (serous cystadenocarcinoma) and SKOV3 (serous papillary cystadenocarcinoma), as well as MR182 (Type II Mature Epithelial Ovarian Cancer cell) isolated from the ascites or tissues of ovarian cancer patients were cultured in RPMI-1640 (BI) replenished with 10% fetal bovine serum (FBS) (BI), 100 μg/ml streptomycin (Sigma), and 100 μg/ml penicillin (Sigma). HO 8910 PM (highly invasive HO 8910, serous cystadenocarcinoma) and the human embryonic kidney (HEK) -HEK-293T cells were cultured in Dulbecco's Modified Eagle's Medium (DMEM) (BI) containing 10% FBS. All cells were cultured at 37 ℃ in a humidified 5% CO_2_ incubator. When the cells in the six-well plate reached a fusion degree of 60-70% per well, Lipofectamine 2000 (Thermo Fisher Scientific) was applied for transient transfection with 50 nM of small RNA oligos (RiboBio, Guangzhou, China) and overexpression and/or shRNA plasmids. These cells were digested and collected after 24 hours of transfection for a series of subsequent assays. Lentivirus expressing miR-222-3p was purchased from Genechem (Shanghai Genechem Co, LTD, China), and miRNA mimics or inhibitors. Gene-specific overexpression vectors, and siRNAs were purchased from RiboBio (Guangzhou, China) The siRNA sequences are listed in Supplementary Table S1.

### Online database analyses

miRDB 26 (http://www.miRdb.org/cgi-bin/search.cgi/), PicTar (https://pictar.mdc-berlin.de/cgi-bin/new_PicTar_vertebrate.cgi/), miRTarBase (http://miRtarbase.mbc.nctu.edu.tw/php/index.php/) and Targetscan 27 (http://www.targetscan.org/vert_72/) were used to predict potential mRNAs that could be targeted by miR-222-3p. Kaplan-Meier (KM) plotter (http://kmplot.com/analysis/) was utilized to compare the overall survival and prognosis differences between the 'PDCD10^high^ expression group' and the 'PDCD10^low^ expression group', and the unpaired two-tailed t-test was used to calculate the p-value. TCGA data portal (http://cancergenome.-nih.gov/) was used to analyze the expression levels of PDCD10 in OC patients. We compared the expression levels of PDCD10 in normal ovary and ovarian cancer using cancer vs. normal analysis. UCSC 28 (https://genome.ucsc.edu/) and JASPAR 29 (http://jaspar.genereg.net/) were used to predict transcription factors that might affect miR-222-3p.

### Functional annotation and pathway analysis of candidate genes

ClusterProfiler and enrich plot packages were used to predict the function of candidate genes. Gene Ontology (GO) and Kyoto Encyclopedia of Genes and Genomes (KEGG) pathway analyses were performed with the standard of *P<*0.05, and the results were downloaded in text format. Gene set variation analysis (GSVA) is an open-source software package for R and can be downloaded from the website, then condensing information from the low and high expression of PDCD10 groups into KEGG pathway.

### RNA isolation and qPCR

The total RNA was isolated from tissues and cell lines using TRIzol reagent (Vazyme) according to the manufacturer's protocol. Reverse-transcribed complementary DNA was synthesized using the GoScript Reverse Transcription System (Promega Corporation, Fitchburg, WI, USA). The All-in-One™ miRNA First-Strand cDNA Synthesis Kit (GeneCopoeia, Rockville, MD, USA) was used to reverse transcription of miRNAs. After the dilution of cDNA with nuclease-free water by 1: 3, qPCR was performed using the Applied Biosystems 7500 Real-Time PCR System and the Go Taq qPCR Master Mix (Promega Corporation, A6001). miRNA was then quantified using the All-in-One™ miRNA qPCR assay kit (GeneCopoeia, Rockville, MD, USA). After the completion of the qPCR procedure, we established a fixed threshold for the circulation threshold (CT) data, and calculated the average CT from the three repeated response wells and from triplicate reaction wells. We chose glyceraldehyde-3-phosphate dehydrogenase (GAPDH) or RNU6-1 (U6) as an internal control (IC) to normalize protein-coding genes or miRNAs' expression levels. The fold-change in the expression of target genes (TGs) and miRNA were calculated by the 2^-ΔΔCt^ method. The primer sequences are shown in** Supplementary Table S1**.

### Protein isolation and Western blot

The total proteins of the transfected HO 8910 PM and SKOV3 cells were lysed with RIPA strong lysis buffer (Beyotime, China) containing a protease inhibitor, 1% PMSF (Roche, Mannheim, Germany), and PIC (Roche, Switzerland). After centrifugation for 15 min, protein concentration in the supernatant was measured using a Pierce^TM^ BCA protein assay kit (Thermo). The supernatant was then mixed with protein loading buffer (NCM Biotech) and boiled at 100 ℃ for 5 min. The same amounts of proteins (30 μg) from all samples were separated by 10% SDS-PAGE (Bio-Rad, USA) and transferred onto a PVDF membrane (Immobilon®-P). GAPDH and *β-*actin were used as loading controls. PDCD10, SNAI2, E-cad, VIM and *β-*catenin used in this study were from following sources: antibodies against PDCD10 and GAPDH were purchased from Abcam and Utibody, respectively (ab110531 and UM4002, respectively); antibodies against SNAI2 and GAPDH were acquired from Affinity (AF4002) and Utibody (UM4001), respectively; antibodies against E-cad, VIM, and *β-*catenin (20874-1-AP, 22018-1-AP and 10366-1-AP, respectively) were obtained from Proteintech. After incubation with the secondary antibody, the proteins were visualized with NcmECL Ultra (A + B) (NCM Biotech) and a chemiluminescence imager (MiNiCHeM).

### Luciferase assay

To construct the dual-luciferase reporter plasmid, PDCD10 luciferase vectors used in this study were cloned into psiTM-Check2-control vector (GenePharma, Shanghai, China) to create a wild-type (WT) psiTM-Check2-PDCD10-3'-UTR, which contained the predicted binding site of miR-222-3p on PDCD10's 3'-UTR (positions 162-168: AUGUAGC). We also cloned the mutant (MUT) plasmid to test the binding specificity. The miR-222-3p binding site was mutated from AUGUAGC to AUCAAGCU. All cloned plasmids were verified by sequencing (TsingKe, China). HEK-293T cells were co-transfected with the WT or MUT of PDCD10 and mimic/NC or inhibitor/NC of miR-222-3p using Lipofectamine 2000 (Thermo Fisher Scientific) transfection system protocol. The miR-222-3p promoter regions containing different SNAI2 binding sites were cloned into pGL3-Basic reporter vectors (Promega, USA). When transfecting HEK-293T cells with luciferase vectors and high-/low-SNAI2, we also co-transfected the cells with a pGL3-basic-control vector as a control. Luciferase activities were tested using a Dual-Luciferase® reporter assay system (Promega, Madison, WI, USA).

### Cell migration assay

To measure the migration rate of HO 8910 PM and SKOV3 cells, we conducted the Tanswell assay according to protocols described elsewhere 30. In brief, small RNA oligos or plasmids were transfected into HO 8910 PM or SKOV3 cells. After transfection for 48 h, the treated cells were digested and resuspended in FBS-free RPMI-1640 medium, then 1×10^5^ cells were counted and inoculated in the upper chamber (about 8 µm in diameter) in the 24-well plates (Corning), and 800 ml of RPMI 1640 containing 20% FBS was added to the lower chamber as a chemoattractant, and cultured at 37 °C in a 5% CO_2_ environment. Cells from the upper chamber migrated across the holes into the lower chamber within 24 h were counted and used as a read-out to quantify cell migration. After 24 h, 4% paraformaldehyde was used to fix the migrated cells for 30 min and dyed with 5% crystal violet for 15 min. A cotton swab was then used to remove the un-migrated cells in the upper chamber. We did three independent trials. The migration of EOC cells in four fields of view was randomly selected and analyzed in an independent trial with 200X magnification under an inverted microscope (Olympus Corp).

### Wound healing assay

We conducted the wound healing assay according to protocols described elsewhere 31. The cells were digested after transfection for 48 h, 4×10^5^ cells were counted and inoculated in 6-well plates. The cells were cultured until a 90%-100% fused cell monolayer was formed. We then scratched the cells in the fused monolayer with a pipette tip causing an experimental injury and created a linear thin scratch 'wound'. Subsequently, the cells were cultured in the serum-free medium for 24 h, and the wound healing was observed and photographed under the optical microscope (Olympus, Tokyo, Japan). We did three independent trials. Finally, images have analyzed the migration in three fields of view that were randomly selected and analyzed in an independent trial with 100X magnification under an inverted microscope (Olympus Corp).

### Haematoxylin and Eosin (HE) staining and immunohistochemistry

HE staining was carried out according to the routine procedure. The paraffin-embedded sections of EOC and normal ovaries were obtained from the Department of Pathology, Central South University. The expression levels of PDCD10 in ovarian tumors/normal ovarian tissues and the section of cancerous tissue of mice were evaluated by IHC using an anti-PDCD10 antibody. In brief, the paraffin-embedded parts were dewaxed, hydrated, and washed. We used a universal two-step assay kit (PV-9000); after neutralization of endogenous peroxidase followed by high-pressure antigen retrieval, the slides were blocked with goat serum at 37 ℃ for 1 h and incubated with the primary antibody for 4 ℃ overnight. The next day, the sections were washed with PBS, and treated with enhancers and enhanced enzyme tags in the universal two-step assay kit. Subsequently, the slides were dyed using the DAB developing kit and counterstained with hematoxylin. Finally, after the slides were dried at 65 ℃, they were sealed and examined under the microscope.

### Cell immunofluorescence

HO 8910 PM cells were transfected with 2 μg control vector or 2 μg PDCD10 overexpression plasmid. After transfection for 48 h, the cells were transferred to 96-well clear-bottom plates (Corning Incorporated, Corning, NY, USA) for high content microscopy. The cells were cultured overnight and washed with PBS preheated at 37 ℃, and then fixed with 4% paraformaldehyde preheated at 37 ℃ for 1 h at room temperature (RT). The cells were permeabilized by treating with 0.5% Triton X-100 and then washed before blocking. Subsequently, cells were blocked in 5% normal goat serum (ZSGB-BIO) for 30-60 min at RT, and then incubated with anti-E-cad and anti-VIM polyclonal antibodies diluted 1:50 or anti-*β-*catenin polyclonal antibody diluted 1:100 in PBS for overnight at 4 ℃. On the second day, the cells were washed with PBS and incubated with the secondary fluorescent antibody (diluted 1:500 with PBS) at 37 ℃ in the dark for 1 h and then stained with DAPI for 1 min. Finally, the cells were imaged using a High-Content Imaging System (PerkinElmer) using the 10× long distance objective. The unpaired two-tailed t-test in GraphPad Prism 8 software used for statistical analysis of fluorescence intensity in the images.

### Chromatin immunoprecipitation (ChIP) assay

The binding affinity of SNAI2 and miR-222-3p was analyzed by the ChIP assay using a kit (Beyotime, Shanghai, China) according to the manufacturer's instructions. We used overexpression (OE-SNAI2 cloned into the FUCGW vector) or SNAI2-shRNA vectors to specifically raise or knockdown SNAI2 levels, respectively. The treated cells (1×10^6^) were lysed, ultrasonic treatment was performed for 48 cycles of 20 s on, 20 s off, and centrifuged to extract the supernatant. The beads were then coupled with the target protein antibody (or IgG). In the IP group, the beads coupled with antibodies were incubated with samples to bind the antibodies to target proteins. We used an antibody against SNAI2 to immune-precipitate SNAI2-chromatin complexes. The beads were then used to separate and wash the target protein and achieved the enrichment of target protein and its binding DNA. Anti-IgG (Santa Cruz, USA) was used as the blank control group to exclude the influence of other factors on Chip assay. The ChIP products were amplified by PCR and then separated on 1.0% agarose gels. The primers for amplification are listed in** Supplementary Table S1.**

### Mouse experiments

To probe the metastasis of tumors* in vivo*, we injected stable cells of 4 different groups into the abdomen of nude mice. Five-week-old female nude mice were used in all tumor xenograft experiments. Approximately 1×10^6^ cells in 200 μL of RPMI 1640 were injected into the nude mice. Mice were euthanized at the end 30 days after cell injection, and the tumors were removed for RNA and protein extraction, HE staining, or immunohistochemical (IHC) staining. We performed all animal care and experiments in strict accordance with the 'Guide for the Care and Use of Laboratory Animals' and the 'Principles for the Utilization and Care of Vertebrate Animals' that were approved by the Animal Research Committee of The Third Xiangya Hospital Cancer Center.

### Statistical analysis

All assays were performed in triplicate. The statistical significance of data differences was calculated using an unpaired two-tailed t-test or two-way ANOVA in GraphPad Prism 8 software. Survival curves were analyzed with the Log-Rank test using the Kaplan-Meier method. The correlation between SNAI2, miR-222-3p, and PDCD10 expression was determined by calculating Pearson's correlation coefficient. The unpaired two-tailed t-test was used to analyze the differences between the two groups. All results were shown as average values ± SEM. *P<*0.05 indicated a statistically significant difference.

## Results

### miR-222-3p is significantly downregulated in EOC tissues and represses cell migration *in vitro*

**Figure 1:**
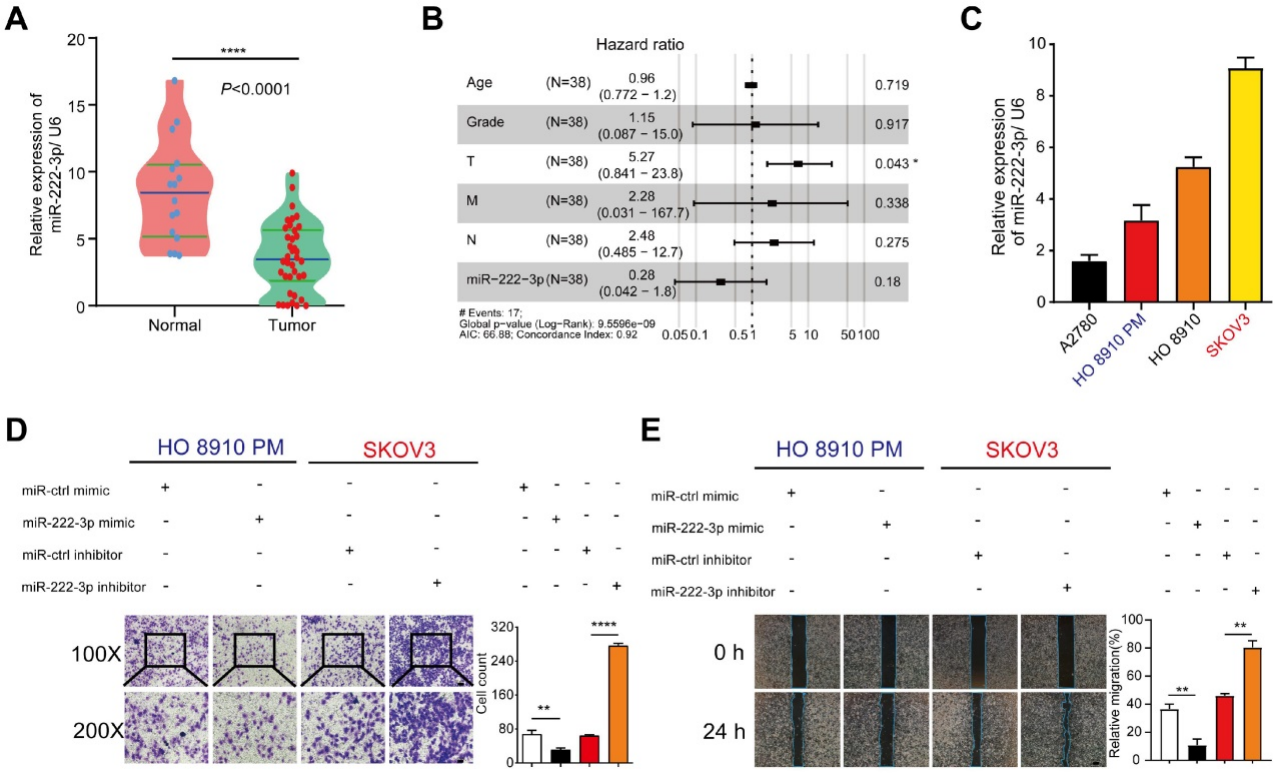
** miR-222-3p is significantly downregulated in EOC tissues and represses cell migration *in vitro*.** (**A**) miR-222-3p was significantly downregulated in EOC tissues (N=16, T=38; *P<*0.0001, revealed by Mann-Whitney test). (**B**) Multiple Cox regression models was used to estimate HRs between miR-222-3p high and low expression levels in each data set within clinical subgroups. (N=38, HR=0.28, *P*=0.18). (**C**) Differential relative miRNA expression of miR-222-3p in four EOC cell lines. (**D and E**) Transwell and wound healing assays revealed that miR-222-3p could inhibit migration of HO 8910 PM and SKOV3 cells in Transwell (**D**) and wound healing assays (**E**). (**D**) 100×microscopic view of the bar, 100 µm. 200×microscopic view of the bar, 50 µm. and the relative migration rate was the number of cells in the 200×microscopic view. (**E**) Bar, 200 µm. All data (C-E) represent the mean ± SD in different assays. **, *P<*0.01; ****, *P<*0.0001, revealed by unpaired two-tailed t-test.

We have previously reported that miR-222-3p could inhibit tumor proliferation by targeting GNAI2 in EOC and could also inhibit cell migration 15. However, the underlying mechanism remained unclear. Herein, we examined the expression level of miR-222-3p in EOC tissues. Significant downregulation of miR-222-3p expression was detected when 38 EOC tissue samples, collected from the department of gynecological oncology, Hunan Cancer Hospital, were compared with 16 healthy tissues (*P<*0.0001; **Figure 1A**). miR-222-3p correlated with a low hazard ratio in 38 EOC tissues (Hazard Ration (HR)=0.28, *P*=0.18). Analysis of these samples also showed that the number of tumor metastases (T) was correlated with a high hazard ratio (HR=5.27, *P*=0.043; **Figure 1B**).

We also conducted a series of *in vitro* experiments to elucidate the role of miR-222-3p in inhibiting cancer cell migration. We first examined miR-222-3p expression levels in four different EOC cell lines (A2780, HO 8910, HO 8910 PM and SKOV3). Cells with miR-222-3p^high^ miR-222-3p^low^ expression are shown in **Figure 1C**, we over-expressed miR-222-3p in HO 8910 PM cells and knocked down miR-222-3p in SKOV3 cells. The miR-222-3p mimic group exhibited a lower migration ability compared with the miR-ctrl mimic group in Transwell and wound healing assays. In contrast, the miR-222-3p inhibitor group showed a higher migration ability compared with that in the miR-ctrl inhibitor group (**Figure 1D and 1E**). These results indicated that miR-222-3p could suppress EOC cell migration.

### miR-222-3p directly suppresses PDCD10 expression by binding to its 3'-UTR and inhibits EOC cell migration *in vitro*

**Figure 2:**
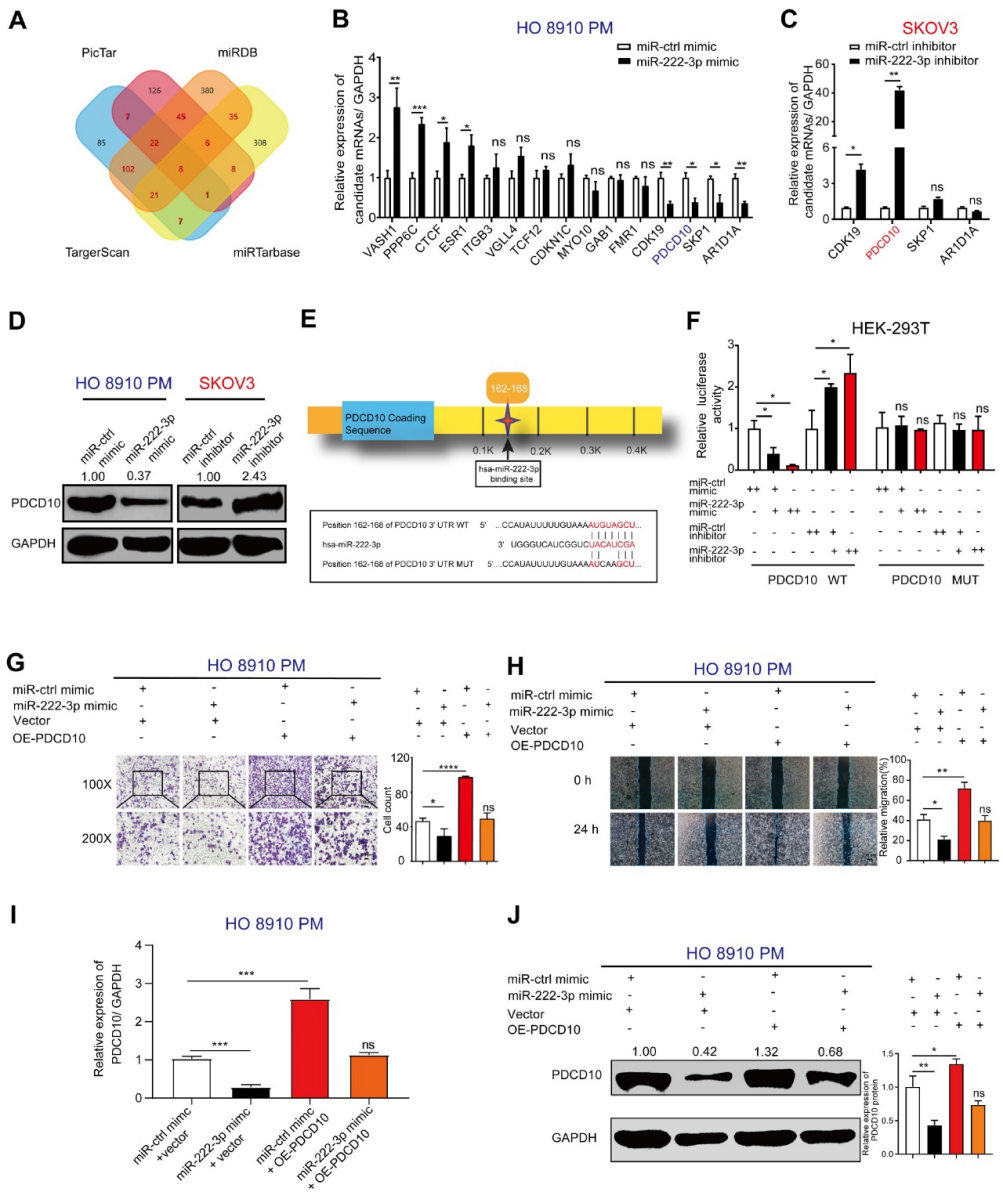
** miR-222-3p directly suppresses PDCD10 expression by binding to its 3' -UTR and inhibits EOC cell migration *in vitro*.** (**A**) A Venn diagram was used to look for candidate genes targeted by miR-222-3p. (**B**) Levels of 15 candidate genes in HO 8910 PM cells transfected with miR-222-3p mimic. (**C**) miR-222-3p inhibitor assay in SKOV3 cells showing four candidate genes with upregulation of two genes, the most significant being PDCD10. (**D**) Western blot analysis of PDCD10 levels in two different EOC cell lines after transfection with miR-222-3p mimic or inhibitor. (**E**) Schematic depiction of the double-strand formation by miR-222-3p with the 3' -UTR of PDCD10. (**F**) Relative luciferase activities in HEK-293T cells co-transfected with a miR-222-3p mimic/inhibitor and PDCD10 WT/MUT. “++” stands for the double concentration. (**G and H**) Transwell and wound healing assays revealed inhibition of migration when HO 8910 PM cells were transfected with miR-222-3p mimic. Recovery assays showed that miR-222-3p suppressed migration of EOC cell lines due to its inhibitory effect on PDCD10 (Left). Cell counting and wound healing were quantified (Right). (**I**) qPCR and (**J**) Western blot analyses of PDCD10 expression levels in HO 8910 PM cells, and Image J calculated the relative expression rate. Data are means ± SEM. Data are from six (B) experiments and representative of three (C and F-J) independent experiments. *, *P<*0.05; **, *P<*0.01; ***, *P<*0.001; ****, *P<*0.0001, determined by unpaired two-tailed t-test.

We used the bioinformatics software, miRDB 26, TargetScan 27, PicTar, and miRTarbase to predict candidate genes that might be targeted by miR-222-3p. As shown in **Figure 2A**, we identified 262 candidate genes by comparing two or more intersecting subsets (**Figure 2A**). Subsequently, we analyzed those 262 genes using GO and KEGG pathway enrichment analyses 32 and found a significant correlation with the pathways of 'miRNA in cancer' through GO analysis (**Figure S1A**). KEGG analysis showed that these genes were also related with 'cell adhesion' (**Figure S1B**). Therefore, we focused on cell adhesion-related genes. Among the 262 genes, 15 genes overlapped between Targetscan, miRDB, PicTar, and miRTarbase analyses (**Figure 2A and 2B**).

We used qPCR to detect the expression levels of the 15 candidate genes in EOC cells, which were transfected with miR-222-3p mimic compared with those transfected with control mimic. As shown in **Figure 2B**, four genes each were significantly upregulated and downregulated, and seven genes had no significant difference between the miR-222-3p mimic and control mimic groups in HO 8910 PM cells. We focused on the four down-regulated genes, CDK19, PDCD10, SKP1, and ARIDA1 and checked their expression after knocking down miR-222-3p using miR-222-3p inhibitor (**Figure 2C**). As displayed in** Figure 2C**, two of the four genes were upregulated in the miR-222-3p inhibitor group. Among them, PDCD10 indicated the most significant change. Therefore, in this study, PDCD10 was selected for further analysis. When PDCD10 protein expression was examined in HO 8910 PM cells by Western blot, decreased levels were observed in the miR-222-3p mimic group, while inhibitor of miR-222-3p increased PDCD10 protein levels in SKOV3 cell (**Figure 2D**).

The presence of only one conserved binding site for miR-222-3p in the 3'-UTR of PDCD10 was predicted by Targetscan (**Figure 2E**). We employed the luciferase assay to determine whether the predicted seed sequence binding site led to miRNA-mRNA interactions. The sequence of PDCD10 3'-UTR including the miR-222-3p binding site was inserted into the psiTM-Check2 luciferase vector. We then co-transfected HEK-293T cells with miR-222-3p mimic/inhibitor and PDCD10 3'-UTR-WT plasmids. As displayed in **Figure 2F**, the heterotopic expression of miR-222-3p dramatically reduced whereas inhibition of miR-222-3p promoted the luciferase activity. Furthermore, we constructed a mutant luciferase vector in which the binding site of miR-222-3p in PDCD10 3'-UTR was mutated to eliminate the interaction between miR-222-3p and PDCD10 mRNA. When the luciferase assay was repeated with the PDCD10 3'-UTR-MUT plasmid, miR-222-3p mimic or inhibitor no longer affected luciferase activity (**Figure 2F**). These results suggested that miR-222-3p could inhibit PDCD10 expression by directly binding to its 3'-UTR.

The above data showed a suppressive effect of miR-222-3p on PDCD10 expression. To ensure whether the effect of miR-222-3p inhibition on EOC cell migration was due to the targeting of PDCD10, we designed the recovery assays as follows: PDCD10 overexpression vector was used to specifically restore PDCD10 expression in HO 8910 PM cells in which PDCD10 was suppressed by the miR-222-3p mimic. The efficiency of the OE-PDCD10 (it was cloned into the pCDH-cmv vector) and control vector (ctrl vector) with GFP fluorescence is presented in **Figure S1C**. **Figure 2G and 2H** show that the restoration of PDCD10 in HO 8910 PM cells completely abolished the migration inhibition effect of miR-222-3p mimic. In Transwell and wound healing assays, the PDCD10 overexpression enhanced the migration of HO 8910 PM cells that had been repressed by miR-222-3p mimic (**Figure 2G and 2H**). Significantly, the recovery of PDCD10 overexpression resulted in a higher percentage of migration compared to the miR-222-3p mimic overexpression group (**Figure 2G and 2H**). As expected, miR-ctrl-mimic and PDCD10 co-transfected groups showed higher PDCD10 mRNA and protein levels than other groups (**Figure 2I and 2J**). The PDCD10 overexpression effectively restored the PDCD10 mRNA and protein levels suppressed by miR-222-3p mimic in HO 8910 PM cells **(Figure 2I and 2J)**. Also, the overexpression of PDCD10 promoted MR182 cell migration in Transwell assay (**Figure S1D**). However, OE-PDCD10 did not significantly promote HO 8910 PM cell proliferation in CCK-8 assay (**Figure S1E**).

Furthermore, two PDCD10 siRNAs were used to specifically inhibit the PDCD10 expression in SKOV3 cells in which the PDCD10 expression was increased by miR-222-3p inhibitor. The efficiency of the PDCD10 expression was analyzed by qPCR and the results showed that PDCD10 siRNA-03 and PDCD10 siRNA-04 were functional siRNAs (**Figure S1F**). As shown in **Figure S1G and S1H**, the two siRNAs significantly abolished the migration increasing role of miR-222-3p inhibitor in SKOV3 cells. In Transwell and wound healing assays, the PDCD10 siRNAs repressed the migration of SKOV3 cells that had been enhanced by the miR-222-3p inhibitor (**Figure S1G and S1H**). **Figure S1I** shows that miR-ctrl-inhibitor and PDCD10-siRNAs co-transfected groups had lower PDCD10 mRNA and protein levels than other groups. The PDCD10 siRNAs effectively suppressed the PDCD10 mRNA and protein expression increased by miR-222-3p inhibitor.

Taken together, these results suggested that miR-222-3p acted as a tumor-suppressor gene to inhibit HO 8910 PM and SKOV3 cell migration by directly suppressing PDCD10 expression.

### miR-222-3p suppresses EOC tumor metastasis* in vivo* by targeting PDCD10

**Figure 3:**
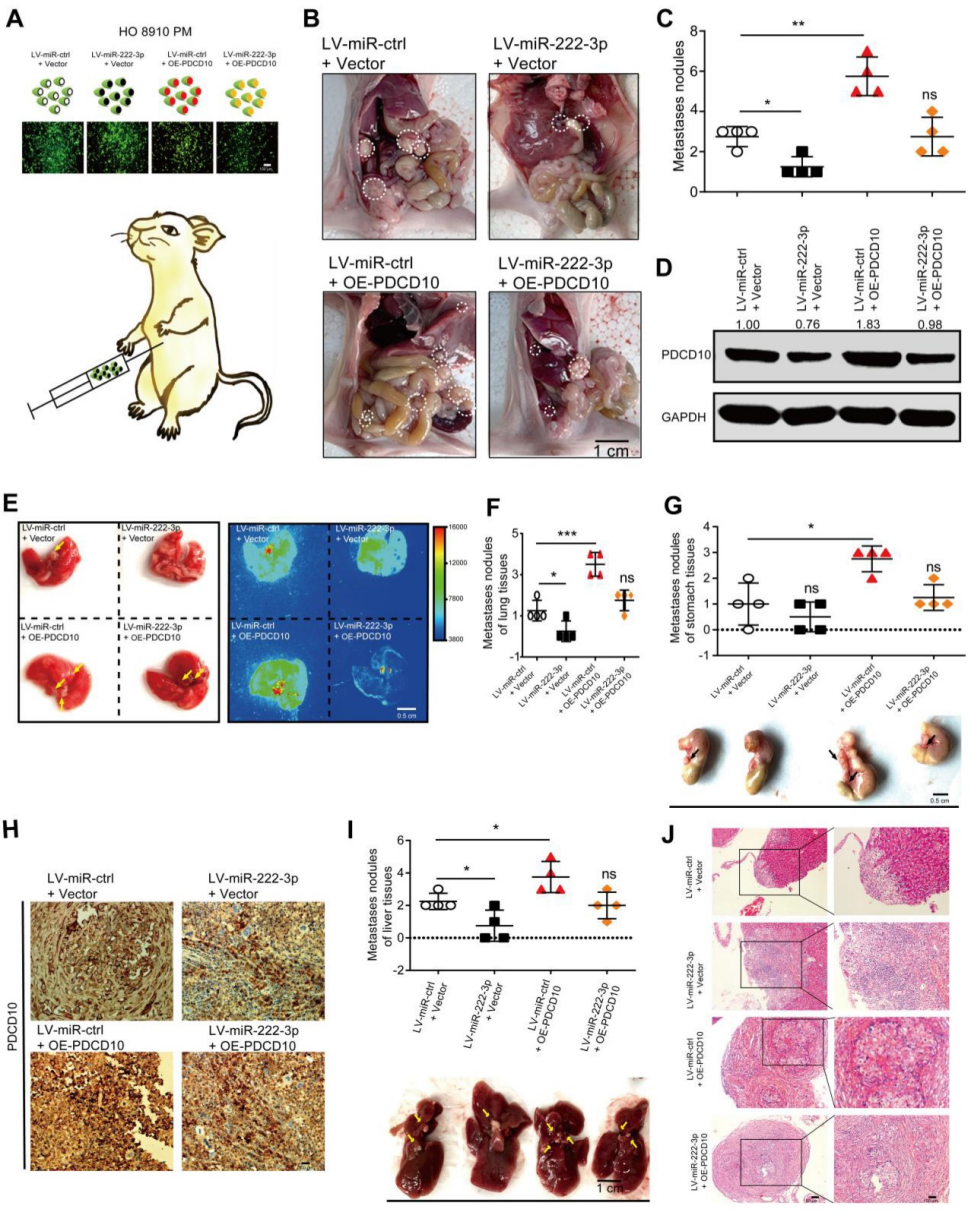
** miR-222-3p suppresses EOC tumor metastasis *in vivo* by targeting PDCD10.** (**A**) Schematic presentation of *in vivo* adhesion for equivalent numbers of GFP-labeled LV-miR-ctrl/LV-miR-222-3p and GFP-labeled ctrl vector/PDCD10 transfected stably in HO 8910 PM cells. Bar, 100 µm. (**B and C**) Representative images and quantification of intraperitoneal metastases in mice implanted intraperitoneally with the same number of HO 8910 PM cells (n= 4 mice per group). Bar, 1 cm. (**D**) Western blot analysis of PDCD10 levels in representative EOC metastatic nodules. (**E**) Representative images and bioluminescence mice images of lung tissue metastatic nodules 5 weeks (wk) after implantation. Bar, 0.5 cm. (**F**) Quantification of metastatic nodules in lung tissues of mice. (**G**) Representative images (Down) and metastatic nodule plots (Up) of mice stomach tissues formed in the restoration group. Bar, 0.5 cm. (**H**) IHC staining for PDCD10 in the stomach tissues of mice 5 weeks after implantation. Bar, 100 µm. (**I**) Representative images (Down) and metastatic nodule plots (Up) of mice liver tissues formed in the restoration group. Bar, 1 cm. (**J**) Representative HE staining of the mice lung tissues obtained from 5 weeks after implantation. Bar, 50 µm (Left) and 100 µm (Right). Data are means ± SEM and are representative of three (C, F, G, I) independent experiments. *, *P<*0.05. **; *P<*0.01; ***, *P<*0.001, determined by unpaired two-tailed t-test.

To verify whether miR-222-3p could inhibit EOC metastasis* in vivo* through targeting PDCD10, we injected GFP-labeled LV-miR-ctrl/LV-miR-222-3p and GFP-labeled ctrl vector/PDCD10 overexpressing stable cells into the abdomen of nude mice to construct the EOC xenograft models (**Figure 3A**). The HO 8910 PM cell group co-transfected with LV-miR-222-3p and ctrl vector showed significantly lower metastasis in the tumor xenograft model than the OE-PDCD10 and LV-miR-222-3p co-transfected group. Restoration of PDCD10 expression reversed the inhibition of tumor metastasis by miR-222-3p (**Figure 3B and 3C**). Western blot analysis of proteins extracted from the tumors showed that the PDCD10 overexpression vector effectively restored its protein levels inhibited by miR-222-3p in EOC metastatic nodules (**Figure 3D**). We also determined the number of metastatic nodules in the lung and abdominal tissues of mice. To monitor the effect of miR-222-3p and PDCD10 expression on tumor metastasis, we used the In-*vivo* imaging system to analyze the images of lung and luminescent tissues. We observed that the number of metastatic nodules in the LV-miR-222-3p and ctrl vector co-transfected groups was significantly lower than the LV-miR-ctrl and ctrl vector co-transfected group, and this phenotype could be reversed in the group co-transfected with LV-miR-222-3p and OE-PDCD10 (**Figure 3E and 3F**). Also, the OE-PDCD10 group restored the metastatic ability of HO 8910 PM-miR-222-3p mimic-cells to a level corresponding to the control (LV-miR-ctrl + ctrl vector) group (**Figure 3E and 3F**). Similarly, using the mice* in vivo* imaging system, we found that the overexpression of PDCD10 in HO 8910 PM-GFP cells resulted in more metastatic nodules on the stomach tissues after 5 weeks. This phenotype could be reversed in the LV-miR-222-3p and OE-PDCD10 co-transfected group (**Figure 3G**). The IHC staining of the metastatic tumor on the stomach tissues of mice detected significantly higher expression of PDCD10 protein in the LV-miR-ctrl and OE-PDCD10 co-transfected groups, and this expression could be reversed in the LV-miR-222-3p and PDCD10 co-transfected group (**Figure 3H**). The liver tissues of mice also showed reduced metastasis in the miR-222-3p-overexpressing group and increased metastasis in the OE-PDCD10 group. However, xenografts with both miR-222-3p and PDCD10 overexpression demonstrated increased metastasis than xenografts with miR-222-3p overexpression alone (**Figure 3I**). H&E staining revealed that tumors of liver tissues from LV-miR-222-3p and PDCD10 co-transfected group displayed a less stroma-rich architecture compared with those from LV-miR-ctrl OE-PDCD10 co-transfected group (**Figure 3J**). Thus, our data showed a negative correlation between the miR-222-3p/PDCD10 regulatory axis and EOC metastasis.

The examination of other organs in mice showed no tumor metastasis in the hearts of mice in the LV-miR-ctrl and OE-PDCD10 co-transfected group in HO 8910 PM cells compared to other restoration groups (**Figure S2A**). However, the LV-miR-ctrl and PDCD10 co-transfected in HO 8910 PM cell group had some difference in the number of metastatic nodules in the spleen tissues compared to other restoration groups of mice, but the *P* value is not achieved significance (*P*=0.1135) (**Figure S2B**).

### miR-222-3p targets PDCD10 to inhibit cell migration via EMT pathway

**Figure 4:**
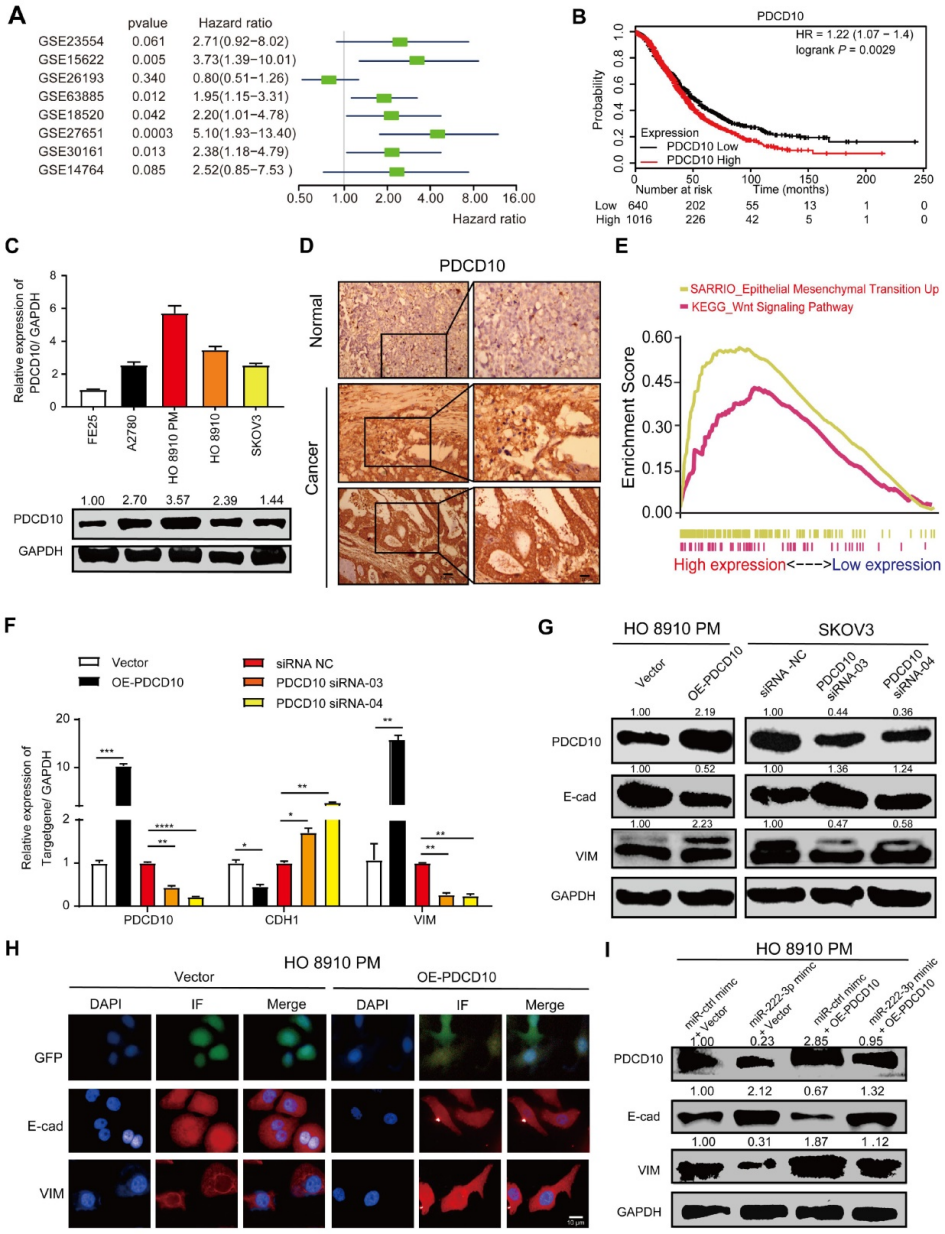
** miR-222-3p targets PDCD10 to inhibit cell migration via EMT pathway.** (**A**) Meta-analysis describing forest plots of PDCD10 expression as a univariate predictor of overall survival. (**B**) Kaplan-Meier curves for overall survival probability in 1656 OC patients with low (n=640) and high (n=1016) PDCD10 expression (analyzed with log-rank test) from KMplot (http://kmplot.com/analysis/). (**C**) qPCR and Western blot analyses of PDCD10 levels in FE25 and 4 different EOC cell lines. (**D**) Representative images of PDCD10 expression in normal and tumor ovary tissues. Bar, 100 µm (Left) and 30 µm (Right). (**E**) Enrichment evaluation within the PDCD10-expressing levels for predicted GSEA results of TCGA reference gene sets for high and low PDCD10 expression groups. Genes expressed in the profile datasets were ranked by fold changes (high-expression/low-expression). GSEA correlation pathways were determined by the given algorithm. Vertical bars along the x-axis of the GSEA diagram represent the positions within the ranked list of genes set in the given sets. Positive and negative GSEA curves mean positive and negative enrichments, respectively. (**F and G**) qPCR and Western blot analyses of PDCD10, E-cad (CDH1) and VIM expression in HO 8910 PM cells (PDCD10-overexpressing groups) and SKOV3 cells (PDCD10-silenced groups). (**H**) HO 8910 PM cells transduced with control vector or PDCD10 (GFP-labeled ctrl vector and PDCD10 all in green) were subjected to immunofluorescence with human-specific E-cad and VIM antibodies (in red). Bar, 10 µm. (**I**) Western blot analysis of PDCD10, E-cad, Vim translation levels in HO 8910 PM cells after treatment with miR-ctrl mimic or miR-222-3p mimic, in the presence or absence of PDCD10. Data are means ± SEM and are representative of three (C and F) independent experiments. *, *P<*0.05; **, *P<*0.01; ***, *P<*0.001; ****, *P<*0.0001, determined by unpaired two-tailed t-test.

PDCD10 was upregulated in various cancers and was shown to play an essential role in tumor signaling 22, 23. Meta-analysis depicting forest plots indicated that PDCD10 mRNA levels correlated with worse patient outcomes in GEO datasets (**Figure 4A**). Besides, we analyzed the correlation between PDCD10 expression levels and survival times of patients in the TCGA database; using the Kaplan-Meier plotter public database, the results showed PDCD10^high^ expression to be frequently associated with poor outcomes of OC patients (**Figure 4B**). We also used the online database of Oncomine 33 to analyze PDCD10 expression in OC patients from Oncomine database. As shown in **Figure S3A**, all types of ovarian adenocarcinomas showed increased expression levels of PDCD10 compared with the normal ovary. This result was validated using 568 OC tissues, in which PDCD10 mRNA levels were significantly upregulated compared with 8 normal ovarian tissue samples in the TCGA database (**Figure S3B**). We have examined both PDCD10 mRNA and protein levels in FE25 and 4 different EOC cell lines (A2780, HO 8910, HO 8910 PM and SKOV3) (**Figure 4C**). We found that PDCD10 mRNA and protein levels in the human fimbrial epithelial cell line were lower than those in the EOC cells. In summary, these results suggested that PDCD10 played an oncogenic role in EOC. Moreover, we randomly selected EOC tissues and ovarian tissues to perform IHC staining for PDCD10 and found its expression to be low in normal tissues but frequently increased in EOC tissues (**Figure 4D**). To identify the pathways that might be involved in PDCD10-mediated ovarian cancer progression, GSEA was performed in the published TCGA ovarian cancer database (n=304). As shown in **Figure 4E**, the PDCD10 levels were positively correlated with the EMT-activated and Wnt-activated gene signatures, suggesting that the EMT (NES=2.83, standardized *P<*0.001) and Wnt (NES=1.69, standardized *P<*0.001) pathways might be involved in the function of PDCD10 (**Figure 4E**).

Since we found a positive correlation between PDCD10, EMT-activated and Wnt-activated gene signatures, the mRNA and protein expression levels of E-cad and VIM in the OE-PDCD10 and PDCD10-siRNAs groups were examined. The mRNA and protein expression levels of E-cad decreased and VIM increased in OE-PDCD10 groups (**Figure 4F, 4G and Figure 3SC**). In contrast, the mRNA and protein expression levels of E-cad increased and VIM decreased in PDCD10-siRNAs groups (**Figure 4F, 4G and Figure 3SC**). We also found that the overexpression of PDCD10 affected either the morphology or the constitutive expression of E-cad and VIM in HO 8910 PM cells (**Figure 4H**). Operetta High-Content Imaging System showed that OE-PDCD10 enhanced the immunofluorescence of proteins associated with the EMT pathway in HO 8910 PM cells (**Figure 4H**). Nuclei intensity per well was analyzed to determine E-cad and VIM protein expression levels in the HO 8910 PM cells by Operetta High-Content Imaging System (**Figure 3SD**). In the recovery assay, the upregulation of PDCD10 in HO 8910 PM cells transfected with miR-ctrl-mimic showed lower E-cad and higher PDCD10 and VIM protein levels compared with other groups (**Figure 4I and Figure 3SF**). However, overexpression of PDCD10 in HO 8910 PM cells after transfection with miR-222-3p mimic resulted in higher E-cad and lower VIM levels compared with miR-ctrl-mimic and OE-PDCD10 co-transfected groups. In summary, our findings suggested that the miR-222-3p/PDCD10 regulatory axis, repressing ovarian cancer cell migration and tumor metastasis, was associated with the EMT signaling pathways.

### miR-222-3p targets PDCD10 to repress cell migration by downregulating the Wnt/*β*-catenin signaling pathway

**Figure 5:**
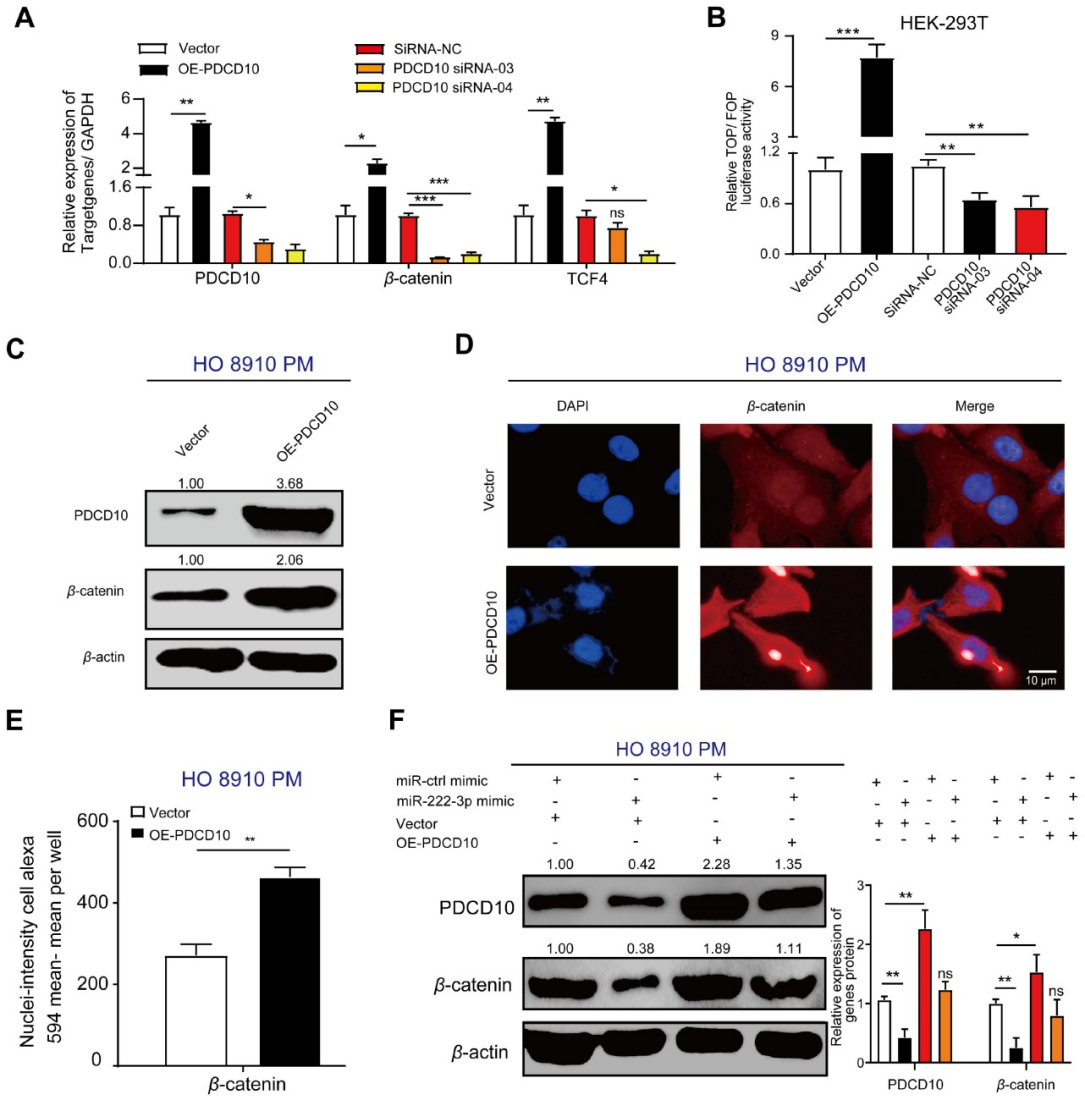
** miR-222-3p targets PDCD10 to repress cell migration by downregulating the Wnt/*β*-catenin signaling pathway. (A)** qPCR analysis of *β*-catenin and TCF4 expression in HO 8910 PM cells (PDCD10-overexpressing groups) and SKOV3 cells (PDCD10-silenced groups). (**B**) Activity of the Wnt/*β*-catenin signaling pathway in HEK-293T cells detected by the TOP flash/FOP flash dual-luciferase reporting system. HEK-293T cells were transfected with TOP flash/FOP flash plasmid and pRL-SV40 vector followed by transfection with or without PDCD10, and siRNA-NC/PDCD10, siRNA-03/PDCD10, or siRNA-04. Firefly luciferase activities were tested by a luminometer, and the activity of the Wnt/*β*-catenin signaling pathway was recorded as TOP/FOP. (**C**) Western blot analysis of PDCD10 and *β*-catenin protein expression following overexpression of PDCD10 in HO 8910 PM cell fractions. *β*-actin was utilized as a marker for the cytosolic fractions. (**D**) HO 8910 PM cells transduced with or without PDCD10 were subjected to immunofluorescence with human-specific *β*-catenin antibodies. (*β*-catenin in red). Bar, 10 µm. (**E**) *β*-catenin nuclei-intensity (detected by Operetta High-Content Imaging System (Perkin Elmer) in HO 8910 PM cell). (**F**) Western blot analysis of PDCD10, *β*-catenin translation levels in HO 8910 PM cells, or after coculturing with miR-ctrl mimic or miR-222-3p mimic in the presence or absence of PDCD10 (Left). The relative expression rate was calculated by Image J (Right). Data (A-B and E-F) represent the mean ± SD in different assays (n=3), *, *P<*0.05; **, *P<*0.01, determined by unpaired two-tailed t-test.

Besides elucidating the regulation of PDCD10 in EOC metastasis via the EMT pathway, the GSEA enrichment analysis revealed that another critical oncogenic pathway, the Wnt/*β-*catenin pathway, was altered in the PDCD10 overexpression group in EOC cells (**Figure 4E**). We examined the expression of *β*-catenin and TCF4 (Wnt target gene) 34 and found the mRNA and protein expression levels of *β*-catenin and TCF4 increased in the OE-PDCD10 group. The mRNA levels of these genes decreased in PDCD10-siRNA groups (**Figure 5A**). A TOP flash/FOP flash dual-luciferase report system was used to further test the activity of the Wnt/*β*-catenin signaling pathway.

The TOP/FOP luciferase activity in HEK-293T cells transfected with OE-PDCD10 group was significantly higher than in the control vector group, and was much lower in HEK-293T cells transfected with PDCD10-siRNA group than in the siRNA-NC group **(Figure 5B).** We detected a significant increase in *β*-catenin protein expression upon PDCD10^high^ transfection in HO 8910 PM cells **(Figure 5C)**. The cell immunofluorescence staining assay also showed a similar result (**Figure 5D**). PDCD10 overexpression in HO 8910 PM cells demonstrated an increase in *β*-catenin immunofluorescence (**Figure 5D**). Using Operetta High-Content Imaging System, nuclei-intensity per well analysis also showed that *β*-catenin protein levels were increased after overexpression of PDCD10 (**Figure 5E**). In the recovery assay, the upregulation of PDCD10 in HO 8910 PM cells after transfection with miR-ctrl-mimic showed higher *β*-catenin translation levels than other groups (**Figure 5F**). However, HO 8910 PM cells co-transfected with miR-222-3p mimic and OE-PDCD10 group had lower *β*-catenin translation levels compared with miR-ctrl-mimic and OE-PDCD10 co-transfected group (**Figure 5F**). These findings indicated that miR-222-3p might also inhibit *β*-catenin expression and repression of the Wnt pathway by targeting PDCD10.

Together, our data indicated a negative correlation between the miR-222-3p/PDCD10 regulatory axis and ovarian cancer metastasis. Also, the miR-222-3p/PDCD10 regulatory axis decreased the migration of EOC cells and tumor metastasis by enhancing the expression of cell adhesion molecules, such as E-cad, and reducing the cellular levels of *β*-catenin.

### SNAI2 inhibits miR-222-3p at the transcriptional level resulting in increased EOC cell migration *in vitro*

**Figure 6:**
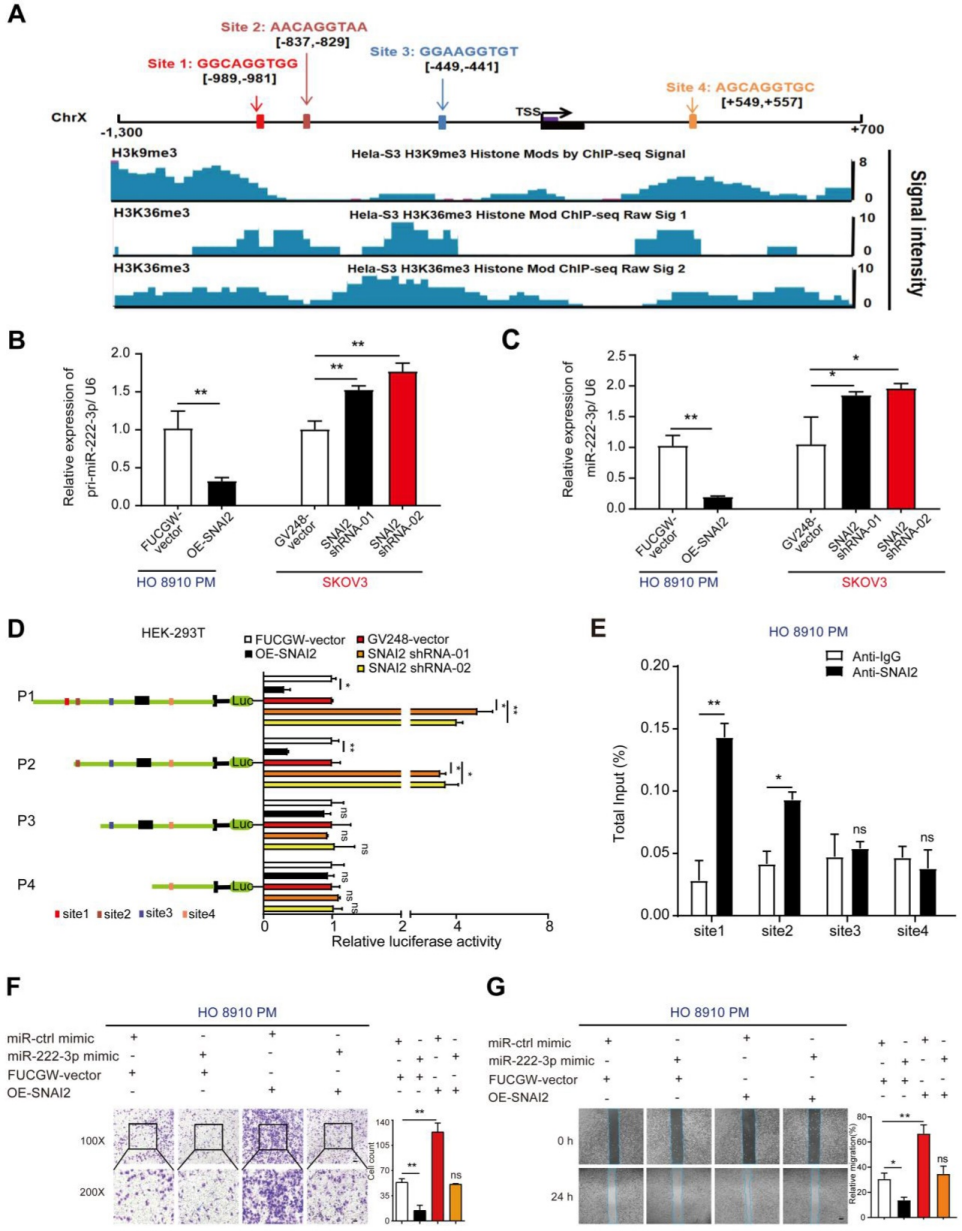
** SNAI2 inhibits miR-222-3p at the transcriptional level resulting in increased EOC cell migration *in vitro*.** (**A**) Schematic presentation of the genomic locations of miR-222-3p and SNAI2's possible binding sites in the promoter region of miR-222-3p host gene chromosome X. The lower part shows the modified state of active histone coordinated from JASPAR ChIP-sequencing data. (**B-C**) Effects of SNAI2 on mature miR-222-3p and pri-miR-222-3p levels. (**D**) Luciferase activities of different miR-222-3p promoter-reporter substructures co-transfected with the SNAI2-overexpressing or SNAI2 shRNA groups. (**E**) ChIP assay for SNAI2's occupancy on the miR-222-3p promoter. (**F**) Transwell (Left) and (**G**) wound healing assays (Left) revealed that SNAI2 increases EOC cell migration when transfected with SNAI2-overexpressing vector in HO 8910 PM cells. Recovery assays indicated that the increase in EOC cell migration by SNAI2 was caused by its suppressive effect on miR-222-3p. Cell counting and wound healing were quantified (Right). Data (B-G) represent the mean ± SD in different assays (n=3), and the relative migration rate was the number of cells in the 200×microscopic view. *, *P<*0.05; **, *P<*0.01, determined by unpaired two-tailed t-test.

As per the JASPAR database, potential SNAI2 binding sites are present on the miR-222-3p promoter. We found an increased level of histone modifications associated with H3K9me3 and H3K36me3 at the promoter of miR-222-3p, which might inhibit the binding of SNAI2 at the miR-222-3p promoter region (**Figure 6A**). Next, we used overexpression or shRNA vectors to specifically raise or knockdown SNAI2's expression, respectively (the efficiencies of OE-SNAI2 and SNAI2-shRNAs are shown in **Figure S4A**), and determined whether SNAI2 could affect the expression levels of mature and pri-miR-222-3p. As displayed in **Figure 6B and 6C**, overexpression of SNAI2 could decrease the expression levels of mature and pri-miR-222-3p in HO 8910 PM cells; knocking down of SNAI2, on the other hand, could increase the mature and pri-miR-222-3p expression levels in SKOV3 cells. These results suggested that SNAI2 could inhibit the transcription of miR-222-3p in EOC cells (**Figure 6B and 6C**). To explore the binding regions of miR-222-3p promoter regulated by SNAI2, various SNAI2 binding sites were cloned into the pGL3-Basic vector for promoter deletion analysis (**Figure 6D**). The data indicated that site1 and site2 were important for the regulation of miR-222-3p transcription by SNAI2, and site3 and 4 had little effect on the regulation of miR-222-3p expression (**Figure 6D**). Furthermore, we performed the ChIP assay to confirm that SNAI2 could bind the promoter of miR-222-3p directly, and observed successful recruitment of SNAI2 by binding sites 1 and site 2 but not binding sites 3 and 4 (**Figure 6E**).

Given that SNAI2 had an inhibitory effect on miR-222-3p expression, we next explored the consequence of SNAI2-driven miR-222-3p overexpression in EOC. To detect the effect of SNAI2 on HO 8910 PM and SKOV3 cell migration, SNAI2-overexpressing and two SNAI2-shRNA plasmids were transfected into HO 8910 PM and SKOV3 cell, respectively. The efficiency of the SNAI2, ctrl-vector, SNAI2-shRNA and GV248-vector (control vector) groups are shown in **Figure S4A**. Transwell and wound healing assays showed that the SNAI2 overexpression promoted EOC cell migration, whereas the miR-222-3p mimic inhibited EOC cell migration. To test whether the improved migration of HO 8910 PM and SKOV3 cells by SNAI2 was related to inhibiting the expression of miR-222-3p, we carried out restoration experiments as shown in **Figure 6F and 6G.** The recovery of OE-SNAI2 in HO 8910 PM cells eliminated the migration inhibition effect of miR-222-3p mimic. In Transwell and wound healing assays, SNAI2 significantly enhanced the migration of HO 8910 PM cells that had been inhibited by miR-222-3p (**Figure 6F and 6G**). At the same time, we also performed recovery assays, which were used to specifically suppress SNAI2 expression in SKOV3 cells. As shown in **Figure S4B and S4C**, restoration of SNAI2 shRNA vectors in SKOV3 completely abolished the migration decreasing effect of miR-222-3p inhibitor. In Transwell and wound healing assays, the SNAI2 shRNA vectors repressed the migration of SKOV3 cells that had been enhanced by the miR-222-3p inhibitor (**Figure S4B and S4C**).

In summary, these results suggested that SNAI2 negatively regulated the transcription of miR-222-3p via specific SNAI2-binding sites in the promoter region of miR-222-3p. Thus, SNAI2 acted as an oncogene to increase HO 8910 PM and SKOV3 cell migration by suppressing miR-222-3p.

### SNAI2 increases cell migration via the SNAI2/miR-222-3p/PDCD10 axis and PDCD10-mediated promotion of EMT and Wnt/*β-*catenin signaling

**Figure 7:**
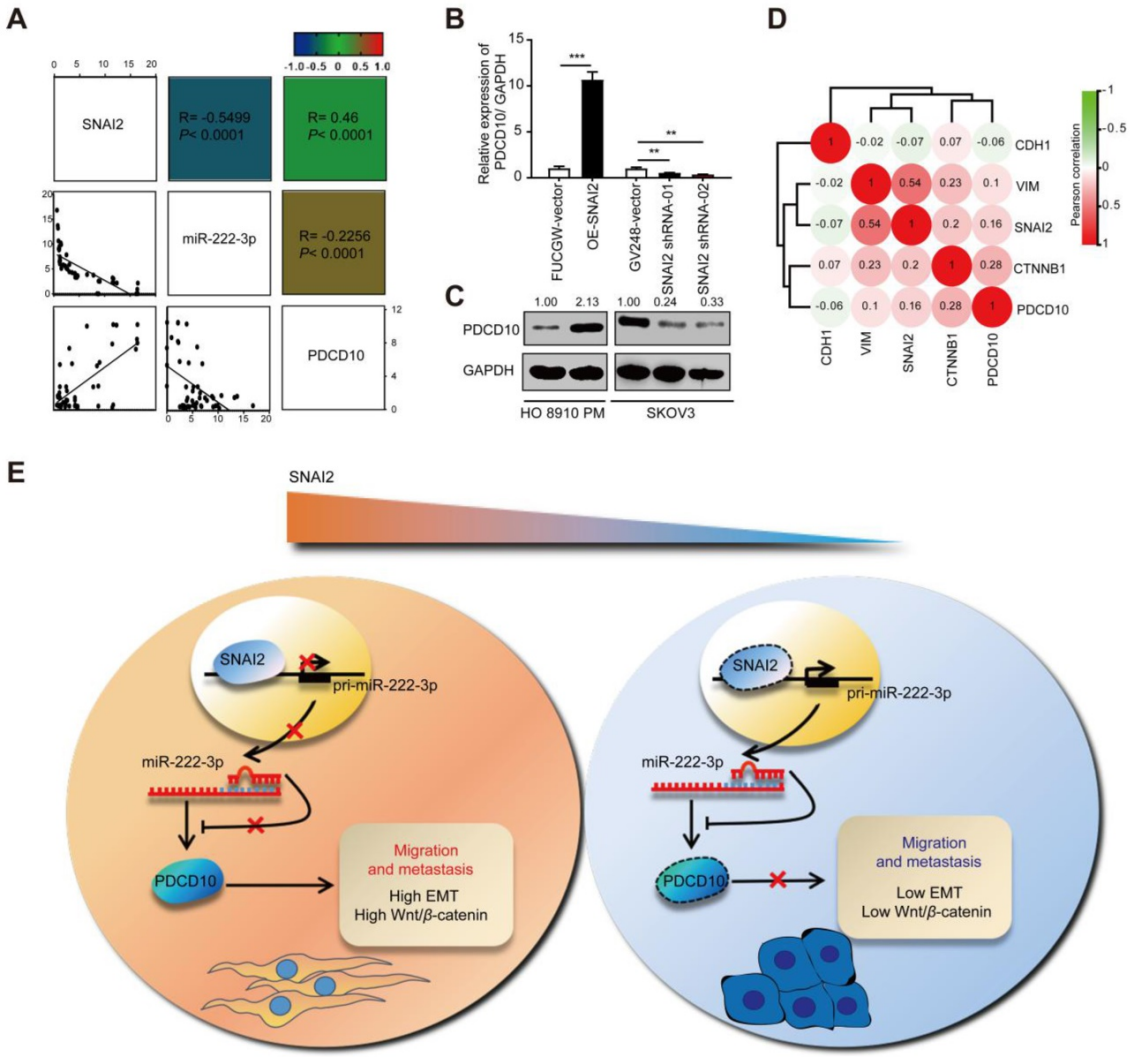
** SNAI2 increases cell migration via the SNAI2/miR-222-3p/PDCD10 axis and PDCD10-mediated promotion of EMT and Wnt/*β-*catenin signaling.** (**A**) Pearson's correlation scatter plots showed the fold changes of PDCD10 mRNA, miR-222-3p miRNA and SNAI2 mRNA levels in EOC tissues (n=38). (**B**) SNAI2 could enhance PDCD10 expression. PDCD10 mRNA expression levels were up-regulated after SNAI2 was overexpressed, but were down-regulated after SNAI2 was knocked down. (**C**) After transfection of SNAI2-overexpressing vectors and SNAI2 knockdown vectors (SNAI2 shRNA-01 and SNAI2 shRNA-02), the expression of PDCD10 was tested by Western blot in HO 8910 PM and SKOV3 cells. (**D**) Spearman correlation analysis of PDCD10, CDH1, VIM, SNAI2 and CTNNB1 levels in OC tissues (n=597) from the TCGA datasets. (**E**) A working model describing the interaction between SNAI2/miR-222-3p/PDCD10 during cancer metastasis. PDCD10 was critical for EOC cell mesenchymal movement and cell survival by maintaining low cell adhesion and high Wnt signaling. SNAI2 regulated PDCD10 by inhibiting pri-miR-222-3p expression and subsequent targeting of genes. SNAI2 regulated PDCD10 by inhibiting miR-222-3p expression, and further activated the downstream EMT signaling and Wnt/*β-*catenin signaling to promote the expression of VIM, and *β-*catenin. These changes contributed to the EOC cell formation and migration, ultimately increasing tumor formation and metastasis. The data (B) represent the mean ± SD in different assays (n=3), **, *P<*0.01; ***, *P<*0.001, determined by unpaired two-tailed t-test.

Since SNAI2 could inhibit miR-222-3p in EOC cells which could target PDCD10, we explored whether SNAI2/miR-222-3p/PDCD10 axis existed in EOC patients and if the expression of PDCD10 was correlated with SNAI2. We performed qPCR analysis of SNAI2, miR-222-3p and PDCD10 expression levels in 38 specimens of EOC patients collected from Hunan Cancer Hospital, and observed a strong correlation between SNAI2 and PDCD10 expression levels (**Figure 7A**). *In vitro* experiments, both qPCR and Western blot results showed that PDCD10 expression level was up-regulated in the OE-SNAI2 group and down-regulated in the SNAI2-shRNA group in HO 8910 PM cells (**Figure 7B and 7C**). Correlations were also observed among PDCD10, SNAI2, CDH1, CTNNB1 and VIM in EOC tissues (n=597) from the TCGA datasets (**Figure 7D**). The expression levels of these genes showed from low to high expression of PDCD10 in the heatmap (**Figure S5**). Therefore, our data suggested that SNAI2 could improve EOC cell migration through the SNAI2/miR-222-3p/PDCD10 axis in EOC.

Our findings are depicted as a cartoon presented in **Figure 7E**. As is well-established, the canonical SNAI2-induced EMT process is through its binding to the promoter of the E-cad gene inhibiting the expression of E-cad to induce EMT. Our findings have shown that high levels of SNAI2 could inhibit the transcription of miR-222-3p causing up-regulated expression of PDCD10, which induced the downstream EMT and activated Wnt/*β-*catenin signaling. In the EOC cells with low SNAI2 expression, high levels of miR-222 could inhibit PDCD10 expression and inhibit EMT and Wnt/beta-catenin signaling pathways.

Our study has unveiled a non-canonical SNAI2-induced EMT pathway, in which SNAI2 represses miR-222 expression and indirectly upregulates PDCD10 expression thus enhancing the Wnt/*β-*catenin pathway and inducing EMT (**Figure 7E**). Therefore, a therapeutic intervention that interrupts the functional SNAI2/miR-222-3p/PDCD10 axis might provide a promising strategy to treat EOC cancer.

## Discussion

In this study, we presented evidence from the large-scale EOC data sets along with *in vitro* and *in vivo* experiments that SNAI2 could inhibit miR-222 expression at the transcription level. Our data also showed that miR-222 could directly repress PDCD10 expression by binding to its 3'UTR. Up-regulation of PDCD10, on the other hand, induced elevated expression of *β-*catenin, a transcriptional repressor of E-cad, which resulted in the promotion EMT and migration of EOC cells. Thus, PDCD10 might be a key regulator of EMT and promotes the EOC invasion-metastasis cascade. We have identified a non-canonical SNAI2-inducing EMT pathway and the findings of our study have significant implications for our understanding of EOC metastasis. A deeper mechanistic understanding of EMT and Wnt/*β-*catenin signaling regulation is needed to improve current anti-metastasis strategies in EOC.

There is a close link between miRNAs and EOC tumorigenic processes. Many miRNAs have significant differential expression and serve as hallmarks at every EOC stage 35. We previously reported that miR-222-3p could inhibit tumor proliferation via targeting GNAI2 in EOC, and lower miR-222-3p expression predicted a worse prognosis 15. Herein, we found that miR-222-3p acted as a tumor-suppressor to inhibit EOC cell migration and tumor metastasis by targeting PDCD10, revealing the significance of miR-222-3p and PDCD10 in EOC. In this context, hepatocellular carcinoma was successfully treated in mouse models by adeno-associated virus (AAV) delivering miR-26a with tumor-inhibitory function 36. A similar strategy could also be applied to target PDCD10 intervention by introducing miR-222-3p and/or a specific small-molecule inhibitor or siRNA 37 for the effective treatment of EOC. It would be worthwhile to explore employing the miR-222-3p /PDCD10 regulatory axis as a therapeutic strategy for EOC.

Several studies have reported that PDCD10 has a variety of biological functions, plays an essential role in tumor signaling and is upregulated in various cancers 22, 23. In our study, PDCD10 was significantly elevated in EOC tissues compared with normal ovarian tissues, and PDCD10^high^ levels predicted lower survival in OC patients. We observed that miR-222-3p was an upstream inhibitor of PDCD10 expression by directly binding to 3'-UTR of PDCD10. Our data revealed that miR-222-3p could target PDCD10 and lead to inhibition of PDCD10 translation, which inhibited EOC cell migration *in vitro* and repressed EOC xenografted tumor metastasis* in vivo*.

The study showed breast cancer metastases occur more frequently in bone, lung, liver and brain tissue, and the tumor metastasis is also organ-specific. Namely, different cancer cells have different propensity to target different organs for metastasis 38. Recent studies have identified a number of genes that mark tissue-specific metastases in breast cancer 39-41. We found no tumor metastasis on the heart and spleen, and the tumor metastasis is more profound in the liver. However, it still needs further study if PDCD10 has an organ-specific role in promoting ovarian cancer metastases.

As gene expression regulators, miRNAs are involved in exquisite regulation of molecular events 42 and coordinate gene expression networks in cancers 35. Several transcription factors (TFs) /miRNA regulatory pairs have been identified, and their critical roles in various cancers have been studied, such as CMYC/miR-1792 and P53/miR-34 43, 44. Also, the downstream target genes of miRNAs have been identified. As for miR-222-3p, several target genes have been reported, such as GNAI2, TIMP3, HIPK2 and PGC-1α 15, 45-47. Herein, we have identified PDCD10 as the downstream target of miR-222-3p, which is downregulated in EOC.

We also examined the upstream regulatory mechanism of miR-222-3p. Employing the UCSC and ENCODE public prediction websites, which predict the transcriptional regulatory factors, we identified SNAI2 as a transcriptional regulator of miR-222-3p. Subsequently, we detected a significant negative correlation between SNAI2 and miR-222-3p in EOC samples. However, the relationship between the SNAI2 transcription factor and miR-222-3p has not been previously reported. Furthermore, we found that SNAI2 interacted with the miR-222-3p promoter and suppressed its transcription. Thus, our results revealed that miR-222-3p was directly inhibited at the transcriptional level by SNAI2, which is a key transcription factor involved in the progression of many cancers 7.

PDCD10 (CCM3) is a member of the CCM family, which includes CCM1 and CCM2 reported to be involved in tumor metastasis, and in many cardiovascular diseases 23. It has been reported that PDCD10 mutations often lead to the development of cerebral vascular lesions or cerebral cavernous malformation 23. Our study found that miR-222-3p/PDCD10 had a tumor-suppressive role by decreasing PDCD10 expression, which in turn inhibited EOC cell migration *in vitro* and tumor metastasis* in vivo*. On the contrary, PDCD10 overexpression regulated the expression of downstream targets and promoted cancer cell migration and tumor metastasis, which suggested a pro-oncogenic role of the PDCD10.

We also found that overexpression of PDCD10 increased cancer metastasis by down-regulating CDH1, enhancing VIM expression, and inducing EMT. Furthermore, overexpression of PDCD10 promoted *β-*catenin which boosted Wnt-mediated cell migration and played an important role in activating Wnt/*β-*catenin signaling, which is regarded as EMT switch of endothelial cells 8. The activation of SNAI2 has been shown to be closely related to the occurrence of EMT 7. Our study identified an essential SNAI2/miR-2223p/PDCD10 regulatory axis in EOC ensuing in increased expression of PDCD10. In breast cancer, TRIM59 has been shown to stabilize PDCD10 by suppressing RNFT1-induced K63 ubiquitination elucidating the exact molecular mechanism governing the TRIM59-PDCD10-RNFT1 axis 48. In our study, we have shown binding of miR-222-3p to the 3'UTR region of PDCD10, suggesting the existence of SNAI2/miR-222-3p/PDCD10/RNFT1 axis in EOC. It is possible that PDCD10 is regulated by a double-positive feedback relationship between SNAI2 and TRIM59, which needs to be explored in the future.

In this study, the feedback loop composed of SNAI2, miR-222-3p and PDCD10 might minimize miR-222-3p expression and thereby boosting PDCD10 expression in EOC cells. As a result, EOC cells become more autonomous, for example, reproducing faster and metastasizing to new microenvironments. Furthermore, this feedback mediation may explain the extensive downregulation of miR-222-3p and the upregulation of PDCD10 in EOC. As direct targets of the SNAI2/miR-2223p/PDCD10 regulatory axis mediated its role in local metastasis. However, there is a multifaceted function in regulating EOC cell migration and tumor metastasis, which will be further subjected to mechanical dissection in follow-up studies in the future.

In this study, we found that PDCD10 could regulate EMT and Wnt/*β-*catenin signaling pathways solely. However, as for other molecules on the molecular determinants governing axis, including SNAI2 and miR-222-3p, it needs further research whether they were involved in indirect regulation.

## Conclusions

Our findings provide evidence for a new regulatory pathway consisting of the SNAI2/miR-222-3p/PDCD10 axis, which upregulates EMT and Wnt/β-catenin to augment EOC cell migration. We believe that these findings have improved our understanding of EOC metastasis significantly. More importantly, both *in vitro* and* in vivo* experiments suggest that PDCD10 could be an effective target for anti-metastasis therapies in EOC.

## Supplementary Material

Supplementary figures and table.

## Abbreviations

EOC: Epithelial ovarian carcinoma
IHC: Immunohistochemical
miRNA: MicroRNA
3'-UTR: 3'-untranslated region
GSEA: Gene Set Enrichment Analysis
E-cad: E-cadherin
IF: Immunofluorescence
TCGA: The Cancer Genome Atlas
EMT: Epithelial-to-mesenchymal transition
KRIT5: Krev-interaction trapped 5
CCM3: Cerebral cavernous malformations 3
GCKⅢ: Germinal center kinase Ⅲ
STR: Short-tandem repeat
DMEM: Dulbecco's modified Eagle's medium
FBS: Fetal bovine serum
NEAA: Nonessential amino acid
CT: Cycle threshold
IC: Internal control
TGs: Target genes
ChIP: Chromatin immunoprecipitation
H&M: Hematoxylin and eosin
HEK: Human embryonic kidney
GO: Gene Ontology
KEGG: Kyoto Encyclopedia of Genes and Genomes
AAV: Adeno-associated virus
I/R: Ischemia-reperfusion
