# Non-canonical signaling pathway of SNAI2 induces EMT in ovarian cancer cells by suppressing miR-222-3p transcription and upregulating PDCD10: Erratum

**DOI:** 10.7150/thno.92973

**Published:** 2024-01-09

**Authors:** Lili Fan, Han Lei, Sai Zhang, Yulong Peng, Chunyan Fu, Guang Shu, Gang Yin

**Affiliations:** 1 Department of Pathology, Xiangya Hospital, School of Basic Medical Sciences, Central South University, Changsha, Hunan Province, China; 2 School of Basic Medical Sciences, Central South University, Changsha, Hunan Province; 3 China-Africa Research Center of Infectious Diseases, School of Basic Medical Sciences, Central South University, Changsha, Hunan Province, China

In the initially published version of this article, there was an issue with the image of E-cad in **Figure 4I.** We have located the original image and made the necessary updates. The corrected **Figure 4I** is provided below. The corrections made in this erratum do not affect the original conclusions. The authors apologize for any inconvenience or misunderstanding that these errors may have caused.

## Figures and Tables

**Figure 4I:**
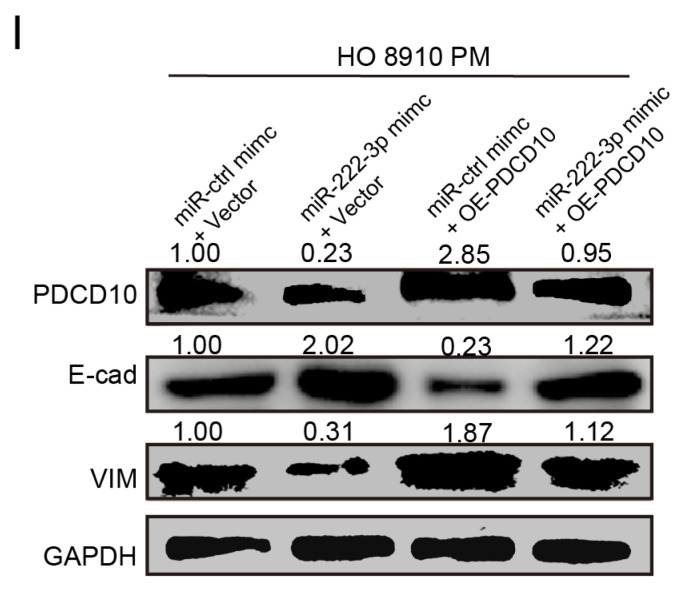
Updated image of E-cad.

