# Supplementary material for: Non-canonical signaling pathway of SNAI2 induces EMT in ovarian cancer cells by suppressing miR-222-3p transcription and upregulating PDCD10
Source: Theranostics. 2020 Apr 27;10(13):5895–913. doi: 10.7150/thno.43198 (PMC7254989; doi:10.7150/thno.43198)
Supplement: Supplementary file 1 — Supplementary figures and table. [file thnov10p5895s1.pdf]

# Supplementary Table

## Supplementary Table S1. Sequences of siRNAs and primers.

| Item | Sequence |
|---|---|
| PDCD10-siR-01 | CCAACCGACTAATTCATCA |
| PDCD10-siR-02 | GAGACCACATCCATGGTTT |
| PDCD10-siR-03 | GAAGCTGAGACCACATCCA |
| PDCD10-siR-04 | CCAGGATGTTGAATGGGAT |
| PDCD10 qPCR primers | F：5’-GCCCCTCTATGCAGTCATGTA-3’ |
| | R：5’-AGCCTTGATGAAAGCGGCTC-3’ |
| SNAI2 qPCR primers | F：5’-GTATCTCTATGAGAGTTACTCCATGCCTG-3’ |
| | R：5’-TTACATCAGAATGGGTCTGCAGATGAGC-3’ |
| pri-miR-222-3p primers | F：5’-CGGCTAGCGCCACCGTAGGAAACAATAATTGGCCATGTGGTCA-3’ |
| | R：5’-GCCGCTCGAGCTTCAACACAACTGCCTACTGCATTCAAG-3’ |
| SNAI2 binding site 1 ChIP primers | F：5’-ACCTACCTACCTATCTACCCCT-3’ |
| | R：5’-AGCTGGATGGAAGGAAGGTC-3’ |
| SNAI2 binding site 2 ChIP primers | F：5’-GTCCAACATTATCAGCTGGGG-3’ |
| | R：5’-ACAGGTAAGAGGTAAAACATGGA-3’ |
| SNAI2 binding site 3 ChIP primers | F：5’-CTTCCACAGAGCCCCTCC-3’ |
| | R：5’-CCATCAGAGACCCAGTAGCC-3’ |
| SNAI2 binding site 4 ChIP primers | F：5’-AGTAAAGAGAACACCAATCCTGT-3’ |
| | R：5’-ACCAAACATCTCAAACACCAAAG-3’ |
| GAPDH qPCR primers | F：5’-GTCGCCAGCCGAGCCACATC-3’ |
| | R：5’-CCAGGCGCCCAATACGACCA-3’ |

Note. F: Forward; R: Reverse.

# Supplementary Figures

**Figure S1 PDCD10 was highly expressed in ovarian tissues and was targeted by miR-222-3p to suppress EOC cell migration. (A)** Rich regulator plot of GO pathway enrichment analysis degree of color represents the P-value; size of the node stands for the number of genes in this item). The 262 genes were significantly correlated with the pathway of 'microRNAs in cancer' in the GO analysis results. (**B)** KEGG pathway classification of 262 candidate genes. The x-coordinate represents the genes labeled in KEGG database, and the ordinate represents the categories of the pathway in KEGG database. **(C**) Effectiveness of the PDCD10 and ctrl vector with GFP fluorescence (Left), and effectiveness of PDCD10 and PDCD10 compared with ctrl vector (Right). (**D**) Transwell assay showed that MR182 cell migration was promoted after transfection with OE-PDCD10 plasmids. (**E**) The viability of HO 8910 PM treated with OE-PDCD10 was assessed by the CCK-8 assay. (**F**) Effectiveness of the PDCD10 and PDCD10 siRNAs compared with ctrl vector and siRNA-NC, respectively. (**G and H)** Transwell and wound healing assays showed that SKOV3 cell migration was promoted after transfection with miR-222-3p inhibitors. Recovery assays demonstrated that the PDCD10 siRNAs repressed the migration of SKOV3 cells that had been enhanced by a miR-222-3p inhibitor (Left). Quantitative diagram of the migration rate (Right). (**I)** qPCR and Western blot analyses of PDCD10 levels in SKOV3 cell. Data are means ± SEM and the relative migration rate was the number of cells in the 200×microscopic view. Data are representative of three (D, F-G) independent experiments. *, $P<0.05$; **, $P<0.01$; ***, $P<0.001$, determined by unpaired two-tailed t-test. Data are representative of three (E) independent experiments. *P*-value determined by two-way ANOVA.

**Figure S2 Tumor metastases in the recovery experiment. (A)** Representative images and bioluminescence images of the mice heart issues without metastatic nodules formed 5 weeks after implantation. Bar, 0.5 cm. (**B**) Representative images of the mice spleen issues (Left). Bar, 0.5 cm. Metastatic nodules from spleen tissues were quantified (Right). All data (B) represent the mean ± SEM on different assays (n= 4), *P*-value determined by unpaired two-tailed t-test.

**Figure S3. PDCD10 is upregulated in OC tissues and correlated with EMT-related genes.** (**A**) PDCD10 levels in the normal ovary, ovarian clear cell adenocarcinoma, endometrioid ovarian adenocarcinoma, ovarian mucinous adenocarcinoma, and ovarian serous adenocarcinoma analyzed by Oncomine database (We used the following filters: gene 'PDCD10', Analysis Type: 'Cancer vs. Normal Analysis', CancerType: 'Ovarian Cancer'). (**B**) Expression of PDCD10 was significantly upregulated in 568 ovarian tumor tissues (Tumor) compared with 8 normal ovarian tissue samples (Normal) in the TCGA profile. ($P<0.0001$, revealed by Mann-Whitney test). (**C**) Total PDCD10, E-cad and VIM proteins were measured by immunoblot and the relative expression rate was calculated by Image J. (**D**) E-cad and VIM nuclei-intensity in per well detected by Operetta High-Content Imaging System in HO 8910 PM cells. (**E**) Summary

immunoblot data (n=3) and PDCD10, E-cad and VIM protein levels were quantified by Image J. All data (C-E) represent the mean ± SEM on different assays (n=3), *, $P<0.05$; **, $P<0.01$; ****, $P<0.0001$, determined by unpaired two-tailed t-test.

**Figure S4. Transfection efficiency of the SNAI2 plasmids and SNAI2 increases EOC cell migration in vitro by suppressing miR-222-3p. (A)** Efficiency of SNAI2, SNAI2 shRNA-01 and SNAI2 shRNA-02 compared with ctrl plasmid and ctrl shRNA-plasmid. (**B and C)** Transwell and wound healing assays showed that SKOV3 cell migration was promoted after transfection with miR-222-3p inhibitors. Recovery assays demonstrated that the SNAI2 shRNAs repressed the migration of SKOV3 cells that had been enhanced by a miR-222-3p inhibitor (Left). Quantitative diagram of the migration rate (Right).

All data (A-C) represent the mean ± SEM on different assays (n=3), *, $P<0.05$; **, $P<0.01$; ***, $P<0.001$; ****, $P<0.0001$, determined by unpaired two-tailed t-test.

**Figure S5. Relationship between the low- and high-expression of PDCD10 groups.** The heatmap showed the expression levels of SNAI2, VIM, CTNNB1, and CDH1 mRNAs expression from low to high expression of PDCD10. The distribution of correlation features was compared to expression of these genes between the low and high expression of PDCD10 groups.

# Supplementary Figures

## Figure S1

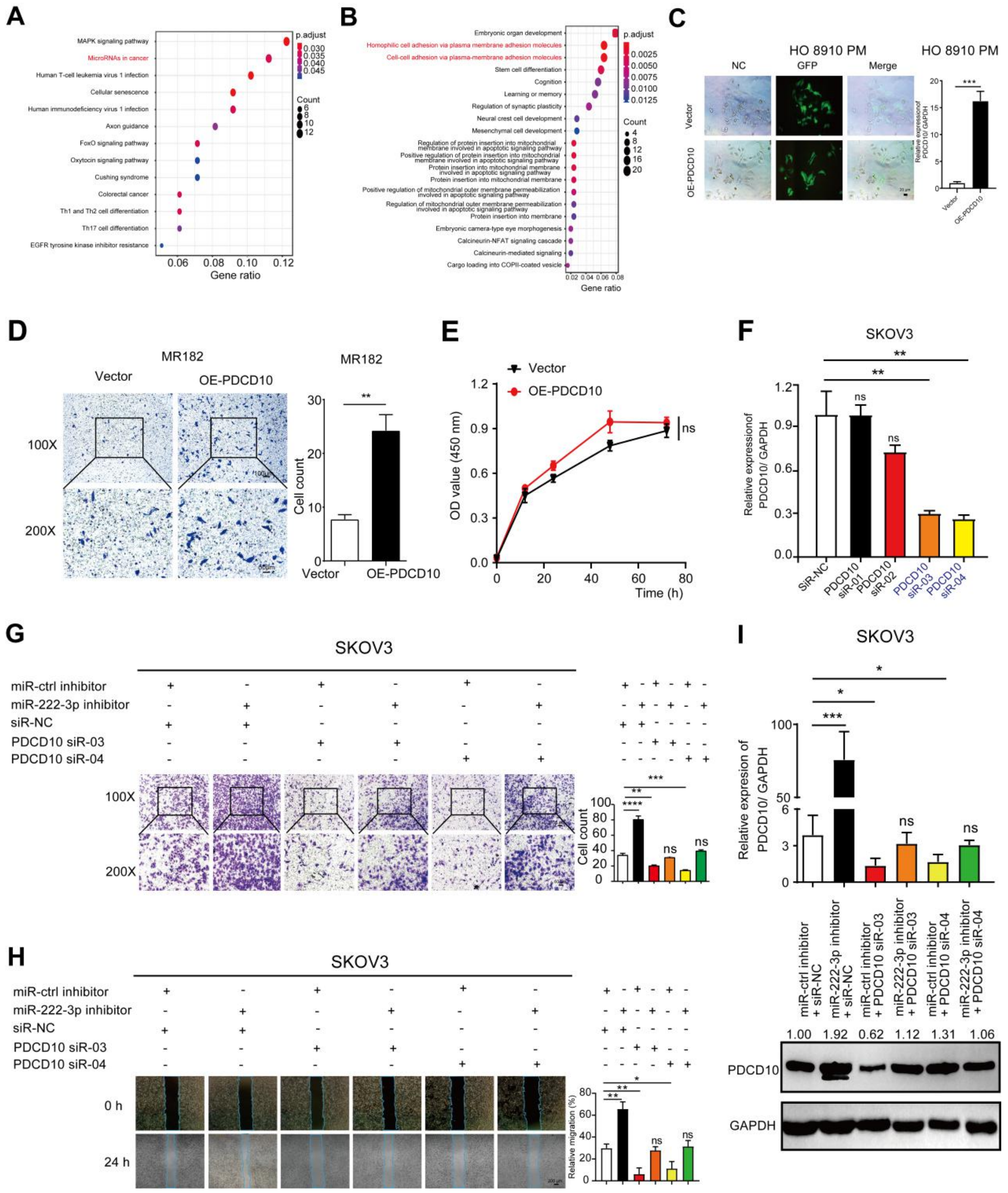

**Figure S2**

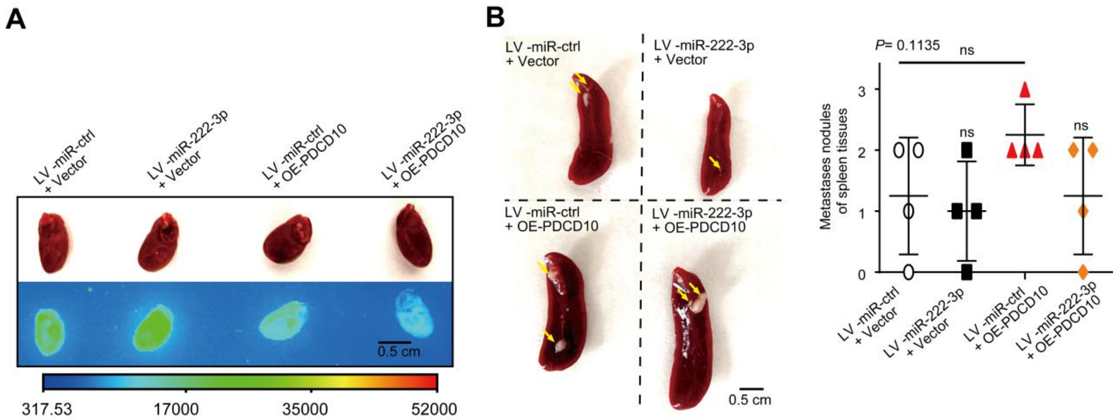
A
LV -miR-ctrl + Vector
LV -miR-222-3p + Vector
LV -miR-ctrl + OE-PDCD10
LV -miR-222-3p + OE-PDCD10
0.5 cm
317.53
17000
35000
52000
B
LV -miR-ctrl + Vector
LV -miR-222-3p + Vector
LV -miR-ctrl + OE-PDCD10
LV -miR-222-3p + OE-PDCD10
0.5 cm
P= 0.1135
ns
Metastases nodules of spleen tissues
0
1
2
3

**Figure S3**

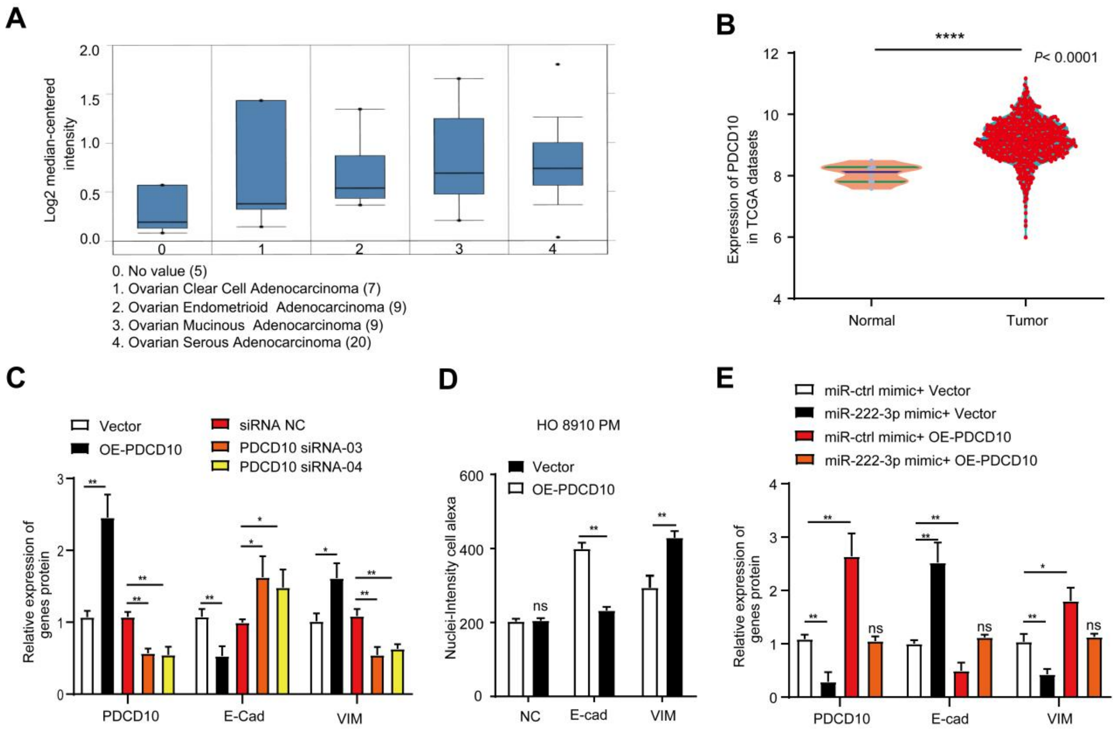
A
Log2 median-centered intensity
2.0
1.5
1.0
0.5
0.0
0
1
2
3
4
0. No value (5)
1. Ovarian Clear Cell Adenocarcinoma (7)
2. Ovarian Endometrioid Adenocarcinoma (9)
3. Ovarian Mucinous Adenocarcinoma (9)
4. Ovarian Serous Adenocarcinoma (20)
B
****
P< 0.0001
Expression of PDCD10 in TCGA datasets
12
10
8
6
4
Normal
Tumor
C
Vector
OE-PDCD10
siRNA NC
PDCD10 siRNA-03
PDCD10 siRNA-04
Relative expression of genes protein
3
2
1
0
PDCD10
E-Cad
VIM
D
HO 8910 PM
Vector
OE-PDCD10
Nuclei-Intensity cell alexa
600
400
200
0
ns
NC
E-cad
VIM
E
miR-ctrl mimic+ Vector
miR-222-3p mimic+ Vector
miR-ctrl mimic+ OE-PDCD10
miR-222-3p mimic+ OE-PDCD10
Relative expression of genes protein
4
3
2
1
0
ns
PDCD10
E-cad
VIM

# Figure S4

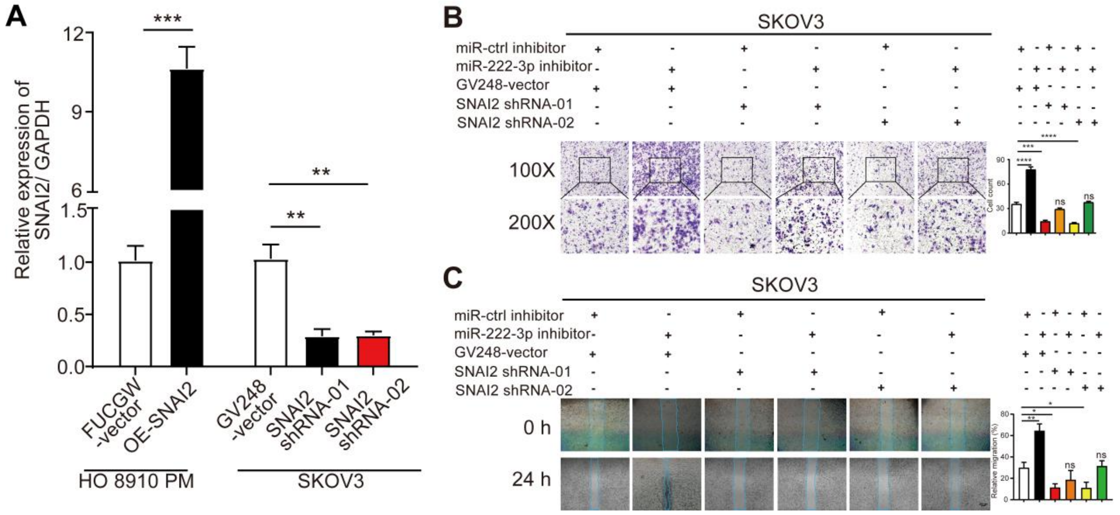
A
Relative expression of SNAI2/ GAPDH
***
**
**
FUCGW -vector
OE-SNAI2
GV248 -vector
SNAI2 shRNA-01
SNAI2 shRNA-02
HO 8910 PM
SKOV3
B
SKOV3
miR-ctrl inhibitor
miR-222-3p inhibitor
GV248-vector
SNAI2 shRNA-01
SNAI2 shRNA-02
100X
200X
Cell count
ns
C
SKOV3
miR-ctrl inhibitor
miR-222-3p inhibitor
GV248-vector
SNAI2 shRNA-01
SNAI2 shRNA-02
0 h
24 h
Relative migration (%)
ns

**Figure S5**

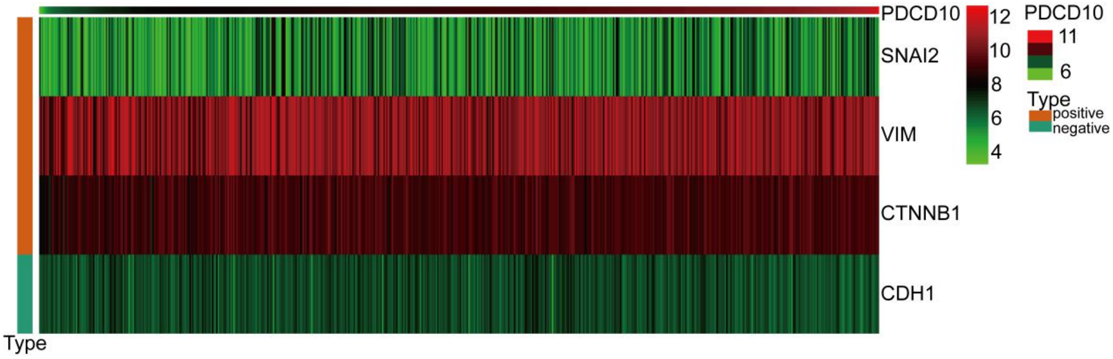
PDCD10
SNAI2
VIM
CTNNB1
CDH1
Type
12
10
8
6
4
PDCD10
11
6
Type
positive
negative
